# Genetic and Phenotypic Characterization of a Large Cohort of Patients with BBS1-Retinopathy

**DOI:** 10.1016/j.xops.2026.101164

**Published:** 2026-03-19

**Authors:** Juan C. Romo-Aguas, Thales A.C. de Guimarāes, Angelos Kalitzeos, Maria Del Pilar Alfaro-Goldaracena, Rebecca A. Baker, Anthony G. Robson, Kaoru Fujinami, Yu Fujinami-Yokokawa, Omar A. Mahroo, Andrew R. Webster, Michel Michaelides

**Affiliations:** 1 UCL Institute of Ophthalmology, University College London, London, UK; 2 Genetics Department, Moorfields Eye Hospital NHS Foundation Trust, London, UK; 3 Department of Ophthalmology, Faculdade São Leopoldo Mandic, Campinas, São Paulo, Brazil; 4 Centro Universitário de Jaguariúna (UniFAJ), São Paulo, Brazil; 5 Centro Universitário Max-Planck (UniMAX), São Paulo, Brazil; 6 International Centre of Eye Health, London School of Hygiene and Tropical Medicine, London, UK; 7 Laboratory of Visual Physiology, Division of Vision Research, National Institute of Sensory Organs, NHO Tokyo Medical Center, Meguro-ku, Tokyo, Japan

**Keywords:** Bardet–Biedl syndrome, BBS1, Syndromic retinitis pigmentosa, Inherited retinal diseases, Gene therapy

## Abstract

**Purpose:**

To analyze the clinical spectrum and natural history of patients with *BBS1*-associated retinopathy.

**Design:**

A single-center retrospective, observational cohort study.

**Patients:**

Molecularly confirmed patients with bi-allelic disease-causing variants in *BBS1*.

**Methods:**

Clinical data were extracted from physical and electronic records. Retinal imaging and electrophysiology were analyzed cross-sectionally and longitudinally. Genetic results were reviewed, and the variants assessed.

**Main Outcome Measures:**

Molecular genetic testing and clinical findings, including best-corrected visual acuity (BCVA), qualitative and quantitative analyses of retinal imaging, and electrophysiology.

**Results:**

Forty-eight patients were identified and assessed longitudinally; 20.8% had isolated retinopathy. The mean age at baseline visit was 28.7 ± 13.7 years, and the mean follow-up was 10.6 ± 7.7 years. The median BCVA was 0.47 logarithm of minimum angle of resolution (LogMAR) (interquartile range 0.3–0.8) at baseline and 1.3 LogMAR (interquartile range 0.7–2.4) at follow-up, with an average decline of 0.05 LogMAR/year. Obesity and polydactyly were the most frequent systemic associations within our cohort. The mean central macular thickness (CMT) at baseline and follow-up was 179.7 ± 43.1 μm and 166.6 ± 50.3 μm. The mean outer nuclear layer thickness (ONLT) at baseline and follow-up was 32.6 ± 21.5 μm and 25.6 ± 22.1 μm. The rate of decline for CMT and ONLT was 4.2 μm and 1.7 μm per year, respectively. When the different genotypes were compared, there was no statistically significant difference between the rates of progression. However, homozygous patients for the most common variant p.(Met390Arg) had an older age of onset and reached legal blindness at an older age compared with the other genotypes. Of the patients who had electrophysiology available, 19 of 27 had rod–cone pattern dysfunction, (6/27) a similar degree of rod and cone dysfunction. One had a cone-rod dystrophy pattern, and the other had only macular dysfunction; both developed a rod–cone pattern during follow-up. Seven *BBS1* variants were reported, and characteristic genotype–phenotype correlations were revealed.

**Conclusions:**

This study is a large cohort with long longitudinal follow-up of molecularly confirmed patients with *BBS1*-associated retinopathy. The ocular and systemic phenotype, detailed imaging, electrophysiological features, and disease progression are described, expanding the spectrum of functional and structural phenotypes related to *BBS1* variants.

**Financial Disclosure(s):**

Proprietary or commercial disclosure may be found in the Footnotes and Disclosures at the end of this article.

Bardet–Biedl syndrome (BBS) is an autosomal recessive condition associated with a wide phenotypic spectrum and is part of a broad group of conditions affecting the primary cilia. Some cardinal features are severe photoreceptor degeneration, postaxial polydactyly, truncal obesity, renal abnormalities, and cognitive/intellectual disabilities.^1^ Retinal dystrophy is the most penetrant and most prevalent feature in patients with BBS, described in >90% of the patients, where the retinal degeneration appears at a very young age with early macular involvement. In previous studies, this pattern would be associated with a cone or cone–rod dystrophy.^2^ The overall prevalence varies within different populations, being most frequent in the regions of the Middle East, Newfoundland, and Kuwait (1:13 500), compared with the United Kingdom (1:125 000) and Europe (1:160 000), where prevalence is lower.^3^

Currently, pathogenic variants in 26 genes have been identified as causing BBS (RetNet, the Retinal Information Network, University of Texas, Houston Health Science Center, Houston, TX, accessed on June 30, 2025, https://retnet.org/). Most pathogenic variants are identified in *BBS1* (Online Mendelian Inheritance in Man #209901) and *BBS10* (Online Mendelian Inheritance in Man #610148)*;* together, they account for nearly half of the BBS cases.^4^ *BBS1,* together with 7 other proteins, form the BBSome complex, an important regulator of ciliary transport. Photoreceptors are highly modified ciliary structures and one of the most metabolically active tissues in the body due to their serial renewal of the outer segments. Previous animal models have identified the role of mutations in *BBS1/BBSome*, affecting the lipid and protein composition of outer segments, which contributes to early functional deficits, as well as subsequent morphological defects.^5^

This study aims to enhance our understanding of BBS associated with the *BBS1* gene through a retrospective analysis of the detailed ocular and systemic phenotype and by examining possible genotype–phenotype relationships within this large cohort.

## Methods

The study protocol adhered to the tenets of the Declaration of Helsinki and received approval from all local ethics committees of the participating institutions and Moorfields Eye Hospital. Informed consent was obtained from all adult patients, whereas informed consent and assent were obtained from parents and children, respectively.

### Patients' Characterization

Patients with molecularly confirmed *BBS1*-associated retinopathy with or without syndromic features of BBS were identified by reviewing the clinical records and genetics databases of Moorfields Eye Hospital. All patients included harbored 2 pathogenic variants in *BBS1* and were under the care of a retinal genetics specialist (M.M., O.A.M., and A.R.W.). Patients with other pathological conditions or questionable diagnoses were excluded from further analysis.

Relevant patient data were retrieved from the electronic and physical health care records and imaging databases. The age of disease onset was defined as the age of the first disease-related symptom(s). Snellen visual acuities were recorded and converted to logarithm of the minimum angle of resolution (LogMAR) for descriptive statistics. For patients who had recorded count fingers vision, hand motion, light perception, and no light perception, the following values were recorded, respectively: LogMAR 2.10, LogMAR 2.40, LogMAR 2.70, and LogMAR 3.0.^6^^,^^7^

Patients were categorized using the World Health Organization visual impairment criteria (based on best-corrected visual acuity [BCVA] of the best-seeing eye), which define no or mild visual impairment as ≤0.48 (6/18), moderate impairment as BCVA >0.48 and ≤1.0 (6/60), severe as BCVA >1.0 and ≤1.3 (3/60), and blindness as BCVA >1.3. Records of visual fields were very limited within our cohort; therefore, we only took into consideration BCVA to classify patients functionally.

### Retinal Imaging

Imaging modalities were obtained with spectral-domain OCT (Heidelberg Spectralis; Heidelberg Engineering, Inc), fundus autofluorescence (FAF, Heidelberg Spectralis and Optos PLC), and ultra-widefield fundus color photography (Optos PLC) in patients with dilated pupils.

Qualitative assessment of color fundus photos, FAF, and OCT was performed using all available data in our center, at baseline imaging and last follow-up. Quantitative analysis was performed in all patients by a single experienced observer (J.C.R.A.). Central macular thickness (CMT), ellipsoid zone (EZ) width (EZW), and outer nuclear layer (ONL) thickness (ONLT) were recorded. Qualitatively, the presence of cystic macular edema (CMO) and epiretinal membrane was noted.

The OCT was scrutinized quantitatively by using digital calipers (Heidelberg Eye Explorer version 2.6.5.0; Heidelberg Engineering), at a 1-μm:1-μm display with maximum magnification, on the transfoveal horizontal line scan, with the foveal reflex used as an anatomical landmark. Ellipsoid zone width was measured when the EZ layer was identified as continuous and extended through the central subfoveal region. The ONL thickness was also measured as the distance between the outer plexiform layer and external limiting membrane, except for cases where there was no clear delineation of the ONL. The area within the hyperautofluorescent (hyperAF) ring at the macula was quantified by tracing the internal border of the ring manually using the software area tools (Heyex version 2.6.5.0; Heidelberg Engineering). We used 55 × 55° images given the large area of some of the rings, which would otherwise not be measurable in the 30 × 30° images. When a double ring was present, the innermost ring was measured. If the ring area was beyond the edge of the FAF image, the scan was excluded. The follow-up mode allowed a more accurate measurement in the longitudinal analysis. The area of definitely decreased autofluorescence (DDAF) was quantified manually in the FAF images by using the area tool and tracing the borders of the lesion. The authors used either 55 × 55 or 30 × 30° images, whichever was acquired in higher quality (i.e., automatic real-time). These methods are described in detail in previous studies.^8^

### Genetic Analysis

A combination of Sanger direct sequencing and next-generation sequencing, including a panel of retinal dystrophy genes, whole exome sequencing, and whole genome sequencing, was used to identify variants in *BBS1*.^9^, ^10^, ^11^ All recruited patients were reassessed for their detected *BBS1* variants (*BBS1*: Refseq Reference: NM_024649.5; NP_078925.3; Emsembl transcript ID: ENST00000318312.12; UniProtKB: Q8NFJ9). Sequence variant nomenclature was obtained according to the guidelines of the Human Genome Variation Society by using Mutalyzer 2.0. In silico molecular genetic analysis was performed according to the previous publication (Table S1, available at www.ophthalmologyscience.org).^12^ Patients' specific genotype is detaild in Table S2, available at www.ophthalmologyscience.org. Classification of all detected variants was also performed based on the guidelines of the American College of Medical Genetics and Genomics.^13^

### Electrophysiology

Pattern and full-field electroretinography (PERG; ERG) were performed with gold foil corneal recording electrodes incorporating the International Society for Clinical Electrophysiology of Vision Standards.^14^^,^^15^ The PERG P50 component was used to assess macular function and the full-field ERG, performed under dark-adapted (DA) and light-adapted (LA) conditions, was used to assess generalized (mainly peripheral) rod and cone system functions.^16^ Some participants were tested with xenon-flash stimuli and others with light-emitting diode-based stimuli. To enable comparison across subjects and timepoints, responses were referenced against locally derived, method-specific reference limits. Amplitudes were expressed as a percentage of the lower age-matched reference limit, while peak times were expressed as an absolute difference in milliseconds from the upper age-matched reference limit. Electroretinography phenotype was classed as cone and rod, cone–rod, or rod–cone according to the relative reduction of the DA 10 ERG a-wave compared with the LA 3 ERG b-wave (20% amplitude difference criterion).

### Statistical Analysis

IBM SPSS Statistics for Macintosh (version 28.0. Armonk, NY: IBM Corp) was used for statistical analysis. Descriptive statistics were generated for continuous variables and categorical variables. Continuous variables were reported as either means with standard deviation (SD) or medians with interquartile ranges (interquartile range) depending on normality testing results. Our threshold of statistical significance was set at *P* < 0.05. Normality test (Kolmogorov–Smirnov) was performed to assess data distribution, and Bland–Altman plots were drawn to assess interocular symmetry for each patient at baseline and follow-up. When symmetry was established, only the right eye (OD) is reported for descriptive statistics.

**Figure 1:**
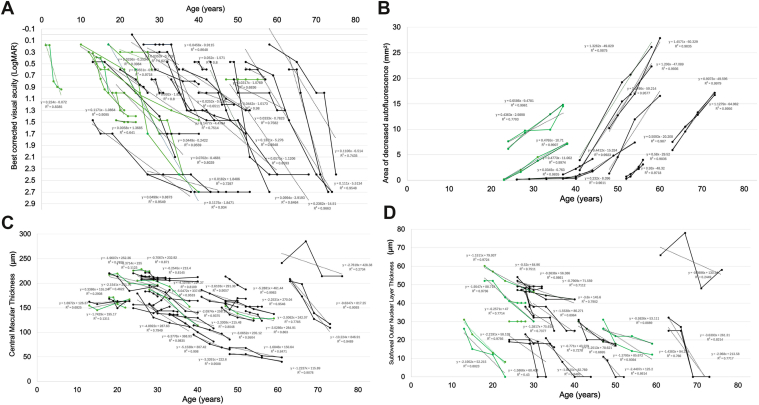
Progression plots. Best-corrected visual acuity **(A)**. Fundus autofluorescence. Area of decreased autofluorescence **(B)**. OCT. Central macular thickness **(C)**, and subfoveal outer nuclear layer thickness **(D)**. Patients who had ≥4 visits with more than a year apart between them were plotted. Black lines represent patients who are homozygous p.(Met390Arg), and green lines depict heterozygous or homozygous patients for p.(Arg160Gln). We can appreciate a progression in all 4 measurements, which happens at a younger age in patients who carry any genotype different from p.(Met390Arg). LogMAR = logarithm of minimum angle of resolution.

Linearity was assessed by performing a regression analysis in patients who had >3 visits, with ≥1 year apart between them (see plots in Fig 1). Linear mixed models were used to analyze the rate of decline of BCVA, EZW, and hyperAF ring area using both eyes. A random intercept was added to the model to account for intereye correlation.

For the assessment of genotype, patients were categorized into 2 groups based on the presence of the most frequent variant compared with other genotypes.

T-test was done for parametric variables' assessment, and Wilcoxon signed-rank test for nonparametric variables. Kaplan–Meier survival curves represent the BCVA loss at different decades.

## Results

### Demographics and Symptoms

Forty-eight patients (96 eyes) from 47 different pedigrees were included in the analysis. All cases identified in this cohort were autosomal recessive with ≥2 disease-causing alleles (bi-allelic). Ethnicity was reported in 28 patients (58.3%). Twenty-one (75.1%) were of White British origin, 5 (17.8%) were of West Asian origin (Turkey), 1 (3.5%) patient was from South Asia (Bangladesh), and 1 (3.5%) patient was of French European origin.

The age at baseline visit was (mean ± SD) 28.7 ± 13.7 years, and the mean age at last visit was 39.6 ± 16.4 years. Twenty-five patients were female (52.1%), and 23 patients were male (47.9%). The mean age of onset was 18.1 ± 11.8 (range: 3–5) years. The first symptom was recorded in 45 (93.8%) of the patients, which was most frequently isolated central vision loss reported in 18 of 48 (37.5%), followed by isolated nyctalopia in 17 of 48 (35.4%) patients. Both nyctalopia with visual field loss and nyctalopia with central vision loss were reported by 3 of 48 (6.3%) patients. Two patients (4.2%) reported a combination of visual field loss and central vision loss, and finally, isolated visual field loss and the combination of all 3 symptoms were reported by 1 patient (2.1%) each. Thirteen (27.1%) patients had photophobia, and 20 (41.7%) had abnormal color vision as found on Ishihara pseudoisochromatic plates.

### BCVA, Refractive Error, and Other Ocular Pathology

At baseline, the median BCVA was 0.47 LogMAR (interquartile range 0.3–0.8). Based on the World Health Organization classification for visual impairment, taking into account visual acuity alone, 28 (58.3%) patients had no or mild visual impairment, 13 (27.1%) patients had moderate visual impairment, 2 (4.2%) patients had severe visual impairment, and 5 (10.4%) patients fulfilled the criteria for blindness. There was a significant correlation between BCVA at baseline and CMT (*r* = –0.485, *P ≤* 00.001), ONLT (*r* = – 0.368, *P ≤* 0.02), and no correlation with DDAF (*r* = – 0.32, *P* = 0.903), age (*r* = 0.216, *P* = 0.140), or gender (*r* = –0.181, *P* = 0.219).

Refraction was available for 38 of 48 (79.2%) patients with a mean spherical equivalent of –2.0 ± 2.1 diopters (+1.00 to –6.50). Twenty-one (43.8%) patients were diagnosed with cataracts in both eyes during their follow-up, with a mean age at diagnosis of 38.9 ± 12.1 years. Six patients had some degree of esotropia (Moorfields Eye Hospital [MEH] 001, 011, 020, 033, 034, and 036). During their follow-up, 2 patients developed choroidal neovascularization (CNV) (MEH 040 – both eyes secondary to idiopathic punctate inner choroiditis, and MEH 046 idiopathic CNV – only left eye [OS]), 2 patients developed a macular hole (MEH 025 and 047), 1 patient had a rhegmatogenous retinal detachment in 1 eye (MEH039), and 1 patient developed papilledema secondary to idiopathic intracranial hypertension (MEH 006). For the statistical analysis, we included data from these eyes up until the occurrence of these conditions.

### Systemic Associations

The World Health Organization body mass index classification was used to determine the patients' weight status.^17^ Body mass indices were available in 31 patients, of whom 22 of 31 (71%) were classified as obese, 8 of 31 (25.8%) as overweight, and 1 (3.2%) patient as having normal weight. The mean body mass index for all patients was 35.7 ± 7.7 kg/m2, while the mean body mass index of the patients in the obesity category was 39.1 ± 6.4 kg/m2.

Polydactyly was the second most common systemic association, affecting 53.3% of the patients. Among those with polydactyly, the presence of an extra digit in 1 limb was reported in 57.7% and, less commonly, in 3 or 4 limbs (7.6% each). Thirty-five percent of the patients had different types of intellectual disability, and renal pathology was present in 18.8% of the patients. A more detailed list of less frequent systemic associations is described in Table 1. Ten (20.8%) patients did not have any systemic conditions and were classified as nonsyndromic.

**Table 1:** Systemic Associations of Patients with *BBS1*-Related Retinopathy

Systemic Associations	N (%)
Body mass index	
Obesity	22/31∗ (71.0%)
Overweight	8/31∗ (25.8%)
Normal	1/31∗ (3.2%)
Polydactyly	26/48 (58.3%)
1 limb	15/26 (57.7%)
2 limbs	7/26 (26.9%)
3 limbs	2/26 (7.6%)
4 limbs	2/26 (7.6%)
Intellectual disability	17/48 (35.4%)
Speech disorder	7/48
Learning difficulties	7/48
Dyspraxia	3/48
Renal abnormalities	9/48 (18.8%)
Chronic kidney disease	4/9 (44.4%)
Kidney cyst	3/9 (33.3%)
Kidney hypoplasia	2/9 (22.3%)
Diabetes mellitus	5/48 (10.4%)
Cardiovascular	3/48 (6.3%)
Reproductive	3/48 (6.3%)
Dental abnormalities (dental crowding)	3/48 (6.3%)
Other	
Systemic hypertension	3/48 (6.3%)
Eczema	3/48 (6.3%)
Psoriasis	3/48 (6.3%)
Hip replacement	3/48 (6.3%)
Hirschsprung disease	2/48 (4.2%)
Asthma	2/48 (4.2%)
Hyperthyroidism	2/48 (4.2%)
Idiopathic intracranial hypertension	1/48 (2.1%)
Osteoarthritis	1/48 (2.1%)
Pancreatic hypoplasia	1/48 (2.1%)
Perthes hip deformity	1/48 (2.1%)
T-cell lymphoma	1/48 (2.1%)
Fibromyalgia	1/48 (2.1%)
Crohn disease	1/48 (2.1%)
Migraine	1/48 (2.1%)

∗ Body mass index (BMI) was available for 31 of the 48 patients in the cohort.

### Interocular Symmetry

Bland–Altman analysis of the CMT, ONLT, and DDAF area showed a good interocular agreement at baseline and follow-up. The mean bias OD – OS for CMT at baseline and follow-up was 0.2 μm (SD 14.5 μm) and 0.2 μm (SD 15.8 μm) and 95% of differences fell between 28.7 and –28.3 μm, and 33.1 μm and –29.1 μm, respectively. The mean bias for ONLT at baseline and follow-up was –0.48 μm (SD 7.3 μm) and 1.3 μm (SD 6.2 μm) and 95% of differences fell between 13.8 and –14.8 μm, and 13.5 μm and –10.8 μm, respectively. Regarding the DDAF mean bias at baseline and follow-up was –1.15 mm^2^ (SD 2.2 mm^2^) and 0.95 mm^2^ (SD 3.5 mm^2^) and 95% of differences fell between 3.3 and –5.6 mm^2^, and 6.1 and –7.9 mm^2^, respectively.

### Fundus Findings

Forty-four patients (91.6%) had a color fundus photo available. Overall, patients exhibited retinal pigment epithelium changes across the fundus, vessel attenuation, with or without bone-spicule-like pigment in the periphery of the retina, in addition to central macular hypopigmentary changes or macular atrophy.

Within our cohort, 34 (70.1%) patients had some degree of macular atrophy, and peripheral lacunae of atrophy were seen in 16 (33.3%) patients, and pigment deposition was observed in 47% of the patients. The presence of macular atrophy, peripheral atrophy, or bone spicule-like pigment did not vary between age groups.

In earlier stages of the disease, fundus color photos showed a predominantly nasal and pericentral retinal pigment epithelium mottling, and patients from later stages showed a tendency to develop bone spicule-like pigment or lacunae of atrophy in the same region first.

### OCT

Forty-seven (97.9%) patients had an OCT scan, and the mean age at baseline was 32.4 ± 15.2 years. The mean CMT at baseline was measurable in 43 of 47 (91.4%) patients with a mean thickness of 179.7 ± 43.1 μm. The ONLT was measurable in 39 of 47 (82.9%) patients with a mean of 32.6 ± 21.5 μm. The EZW was measurable only in 3 (6.3%) patients (MEH 021, 035, 041) at baseline who had a continuous EZ line in the subfoveal region; many patients within our cohort had some remnant of EZ outside the foveal region. The mean EZW in those 3 patients was 1053 μm. Twenty-four (50%) of the patients had epiretinal membrane at baseline, and none of the patients presented with CMO. However, 2 patients developed intraretinal fluid during the follow-up due to CNV (MEH 040 and MEH 046), and 1 patient due to a central retinal vein occlusion (MEH 046).

**Figure 2:**
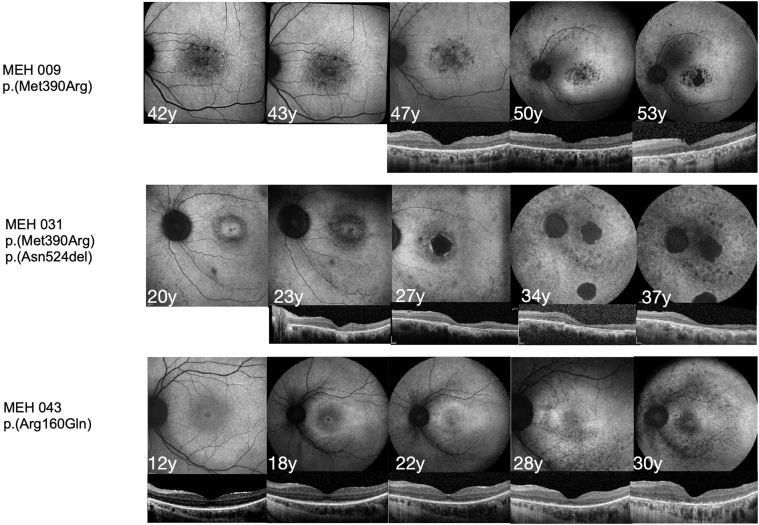
Longitudinal multimodal imaging. Example of fundus autofluorescence images and optical coherence foveal scans comparing 3 patients with different variants who had a follow-up of >10 years. All 3 patients showed early hypoautofluorescence in the central macula which progressed toward a patch of decreased autofluorescence. In addition, the paramacular area shows autofluorescence decline encroaching toward the central macula. MEH = Moorfields Eye Hospital.

**Figure 3:**
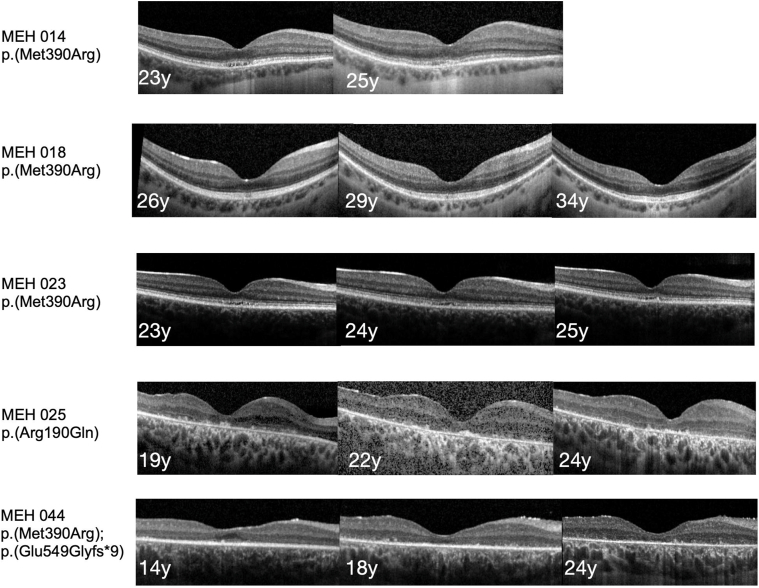
OCT examples of different genotypes. Patients who carry the homozygous p.(Met390Arg) genotype show the following early macular findings: a thin outer nuclear layer, inner/outer segment junction becomes irregular in the subfoveal region extending toward the periphery, and retinal pigment epithelium hypertransmission. In the examples from patients with heterozygous genotype and homozygous p.(Arg160Gln), a complete loss of outer retina at an earlier age can be appreciated. MEH = Moorfields Eye Hospital.

Patients who underwent OCT scans earlier in the disease showed focal irregularities at the level of photoreceptor outer segments and focal thinning of the EZ in the central subfoveal region, and the rest of the EZ extended to the length of the scan (Figs 2 and 3). Later in the disease, in addition to the changes mentioned above, EZ/outer retina loss progressively appears centripetally (Figs 2 and 3). OCT in patients with advanced disease showed a complete loss of the outer retina with a preserved inner retina in most cases (Figs 2 and 3).

### FAF

All patients had short-wavelength FAF images at baseline, with only 2 (4.16%) patients (MEH 018, MEH 046) having a measurable hyperAF ring, with a mean area at baseline of 31.1 mm^2^. Twenty patients (41.6%) showed hypoautofluorescent (hypoAF) peripheral circular patches of atrophy, and 30 (62.5%) patients exhibited decreased area of autofluorescence in the macula. Twenty-two (45.8%) had measurable DDAF area in the macula, with a mean size of 1.5 ± 2.1 mm^2^ (OD) and 2.5 ± 3.5 mm^2^ (OS).

Qualitatively, the initial findings were focal areas of hypoAF in the central macula surrounded, in some cases, by a hyperAF. In addition, patients tended to develop areas of hypoAF in the pericentral and nasal retina later in the disease, which evolved into areas of DDAF. In the late stages, there was a generalized reduction of signal with widespread hypoAF and a significant area of DDAF involving the central macula (Fig 2).

### Electrophysiology

Twenty-seven participants underwent International Society for Clinical Electrophysiology of Vision standard electrodiagnostic testing. Electroretinography amplitudes and peak times demonstrated high concordance between the right and left eyes, with linear regression indicating an approximately 1:1 relationship (component specific *r*^*2*^ ≥ 0.92, slope between 0.91 and 1.06). Due to the interocular symmetry, only OD values are described.

**Figure 4:**
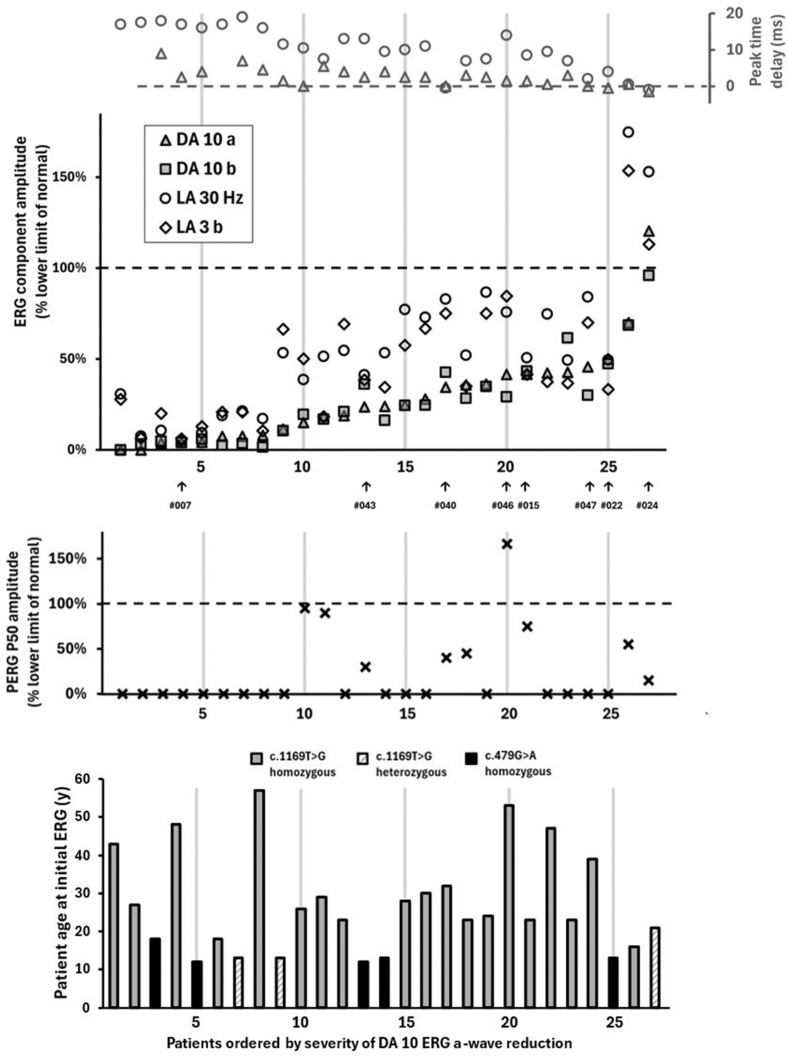
The main ISCEV Standard ERG component measurements are plotted for each participant at initial visit, arranged in ascending order of DA 10 ERG a-wave amplitude. The ERG amplitudes are expressed as a percentage of the lower age-matched limit of the method-specific reference range. Peak times (secondary y-axis) are expressed in millisecond difference from the upper age-matched limit of normal. Arrows indicate patients who were followed up with repeat testing (see Fig 2). The middle chart shows PERG P50 amplitude at baseline for the same patients, expressed as a percentage of the lower limit of the reference range. The lower chart shows patient age at initial ERG, with the genotype highlighted. DA = dark-adapted; ERG = electroretinography; ISCEV = International Society for Clinical Electrophysiology of Vision; LA = light-adapted; PERG = pattern electroretinography.

Patients were aged between 12 and 57 years at initial ERG. Patients homozygous for the c.479G>A, p.(Arg160Gln) variant were significantly younger at initial ERG (median age 13, n = 5) compared with those who were homozygous for the c.1169T>G, p.(Met390Arg) variant (median age 28, n = 19) (*P* = 0.0001, 2-tailed Mann–Whitney test). Severity of retinal dysfunction at initial ERG did not show any obvious correlation with age (Fig 4).

Electroretinography at baseline revealed a spectrum of retinal dysfunction (Fig 4). All but 2 patients presented with a rod–cone pattern of dysfunction (19/27, 70%, “RCD”) or a similar degree of rod and cone dysfunction (6/27, 22%, “R = C”). Retinal dysfunction ranged in severity from mild to severe, with no clear relationship with age. Pattern ERG P50 was undetectable (18/27, 67%), subnormal (6/27, 22%), borderline (2/27, 7%), or normal (1/27, 4%). The patients with the most severe ERG reduction (DA and LA ERGs <33% of normal amplitude) all had undetectable PERG P50. However, for other patients, PERG P50 reduction did not reflect the degree of generalized retinal involvement, for example, MEH 046 and MEH 047 have similar generalized retinal involvement but have normal and undetectable PERG P50, respectively.

Two patients had less typical electrophysiological findings. Patient MEH022 initially presented with a cone–rod pattern of dysfunction with marked macular involvement. Patient MEH 024 showed no ERG evidence of generalized retinal dysfunction at baseline but had severe PERG reduction in keeping with macular dysfunction. Both patients still progressed to a rod–cone pattern of dysfunction, evident from the ERG at follow-up.

### Molecular Genetics

**Figure 5:**
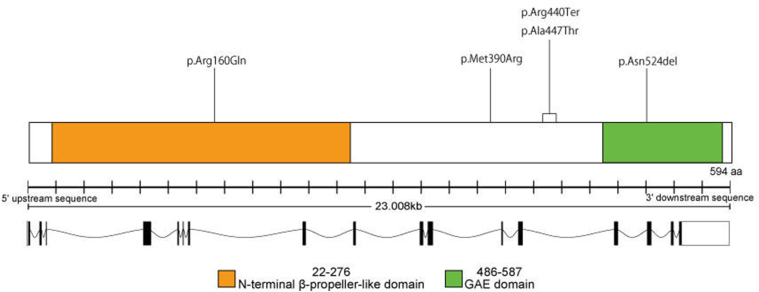
Schematic representation of the BBS1 gene (RefSeq: NM_024649.5) and protein domains indicating the positions of identified variants. The upper panel depicts the BBS1 protein structure, highlighting the N-terminal β-propeller-like domain (residues 22–276) and the gamma-adaptin ear (GAE) domain (residues 486–587). The positions of the missense, nonsense, frameshift, splice-site, and in-frame deletion variants identified in the present study are indicated: p.(Arg160Gln), p.(Met390Arg), p.(Arg440Ter), p.(Ala447Thr), and p.(Asn524del). Two variants, p.(Arg160Gln) and p.(Ala447Thr), are located at exon boundaries and are predicted to result in splice-site alterations. The lower panel shows the genomic organization of BBS1, spanning approximately 23.0 kb, with exons represented as black boxes and introns as connecting lines. Variants are mapped according to their respective positions within the gene and protein.

In total, 7 *BBS1* variants were identified, comprising 3 splice-site alterations, 1 stop-gain, 1 frameshift, 1 missense, and 1 in-frame deletion (Fig 5 and Table S1). Forty-two patients were homozygous for the identified variants, whereas only 6 harbored heterozygous variants. All variants had been reported previously; 3 were classified as pathogenic and 4 as likely pathogenic.

The most prevalent variant was c.1169T>G, p.(Met390Arg), a missense change located in exon 12, detected in 41 patients (78/92 alleles; 84.8%). The second most prevalent variant was c.479G>A, p.(Arg160Gln), a splice-site alteration located at the exon 5 boundary, identified in 6 patients (11/92 alleles; 12.0%). Two patients carried an in-frame deletion, c.1570_1572del, p.(Asn524del) (2/92 alleles; 2.17%). Four variants were detected in single patients: c.951 + 58C > T, p.(Gly318ValfsTer61),^18^ located in intron 10, which creates a cryptic splice-donor site resulting in the inclusion of part of intron 10 and a premature stop codon; c.1318C>T, p.(Arg440Ter), a stop-gain variant in exon 11; c.1339G>A, p.(Ala447Thr), a splice-site alteration at the exon 13 boundary; and c.1643dup, p.(Glu549GlyfsTer9), a frameshift variant in exon 16.

### Longitudinal Analysis

Forty-seven (97.9%) patients had records available for longitudinal assessment with a mean follow-up of 10.6 ± 7.7 (2–30) years. The median BCVA at last visit was 1.3 LogMAR (interquartile range 0.7–2.4), with an average rate of visual loss of 0.05 LogMAR/year. At the last visit, based on visual acuity, 10 (20.8%) patients had no or mild visual impairment, 11 (22.9%) patients had moderate visual impairment, 2 (4.2%) patients had severe visual impairment, and 24 (50%) patients had complete vision loss.

**Figure 6:**
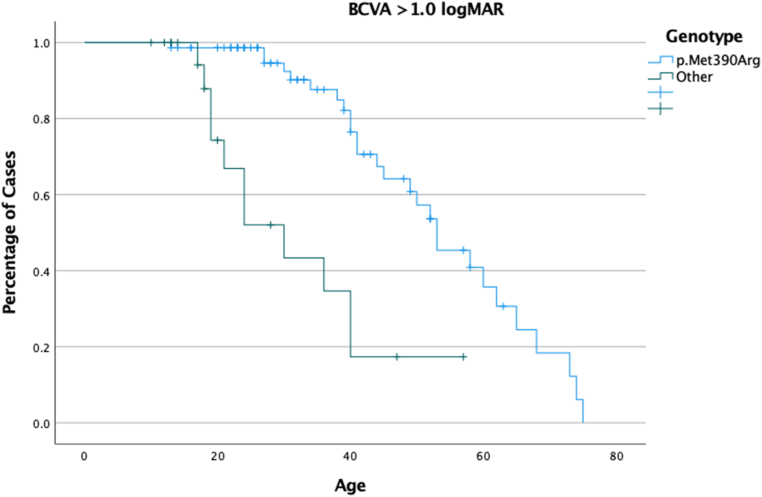
Kaplan–Meier survival analysis showing the percentage of patients with BCVA of ≤1.00 LogMAR (6/60). A log-rank analysis was performed by comparing the most common genotype (homozygous p.(Met390Arg) - blue line) and any other genotype (heterozygous or homozygous p.(Arg160Gln) - green line). Fifty-percent of each group will reach legal blindness by the age of 53 and 30 years, respectively (*P ≤* 0.001). BCVA = best-corrected visual acuity; LogMAR = logarithm of minimum angle of resolution.

Kaplan–Meier survival analysis showed that by the age of 39, 25% of the patients have ≥1.0 LogMAR (6/60), with an increase to 50% at 50 years of age. By comparing the genotypes, we found that patients who were homozygous for p.(Met390Arg) showed that by the age of 53 years, 50% will have a BCVA of 1.0 LogMAR, similar to the entire cohort. On the other hand, patients with other genotype combinations showed that by the age of 30, 50% of them will have a LogMAR of ≥1.0. (log rank, *P ≤* 0.001) (Fig 6).

Forty-three (89.5%) patients had longitudinal OCT data available with a mean time of follow-up of 6.2 ± 4.2 years. Central macular thickness at follow-up was available for 42 of 43 (97.7%) patients with a mean of 166.6 ± 50.3 μm, with a decline of 4.2 μm per year. Thirty-eight (88.4%) patients had a measurable ONLT at follow-up with a mean of 25.6 ± 22.1 μm, with a decline of 1.7 μm per year. Because EZW was measurable in only 2 patients at follow-up, it was not possible to calculate a rate of change over time. There was a statistical difference between the baseline and follow-up measurements of CMT (*P ≤* 0.001) and ONLT (*P ≤* 0.001).

Two patients (4.16%) of the 43 patients at baseline had a measurable hyperAF ring. The mean hyperAF diameter at last follow-up was 9.9 ± 7.9 mm^2^. A rate of progression for hyperAF ring was not calculated because there were only 2 patients with a measurable ring area. At the last visit, 21 (43.8%) patients had FAF images with measurable DDAF, with a mean time of follow-up of 7.7 ± 4.7 years. The mean size was 6.2 ± 7.1 mm^2^ (OD) and 6.7 ± 8.3 mm^2^ (OS), and the rate of change over time was 0.675 mm^2^ per year.

Eight patients underwent repeat electrophysiology testing over periods of 4 to 19 years. There was evidence of progressive retinal dysfunction in all patients, with 7 of 8 showing amplitude reduction of LA and DA ERGs (see Fig S1, available at www.ophthalmologyscience.org), and 2 of 8 showing worsening of LA 30 Hz peak time at follow-up (MEH047 and MEH022, worsening by 3 ms and 10 ms after 9 and 17 years, respectively). The only 2 patients monitored with normal or relatively preserved PERG at baseline (MEH 046, age 53; MEH 015 age 23) had undetectable P50 components after 11 and 7 years, respectively. The patient with a cone–rod pattern of dysfunction at initial ERG (MEH 022, age 13) progressed to a severe rod–cone pattern of dysfunction after 17 years. The patient with preserved rod and cone system function initially (MEH 024) also showed progression, with DA ERGs becoming mildly subnormal and LA ERGs reducing significantly without delay after 4 years, in keeping with a loss of rod and cone photoreceptor function.

### Genotype–Phenotype Correlations

To assess if there was a statistical difference between BCVA, OCT measurements, DDAF area, age at baseline, and age of onset, the cohort was split into 3 groups: patients homozygous for p.(Met390Arg) (n = 37), patients homozygous for p.(Arg160Gln) (n = 6) and compound heterozygous (n = 5). One-way analysis of variance was performed, showing a statistically significant difference for age of onset (F = 3.9, df = 2, *P* = 0.029) and age at baseline (F = 7.4, df = 2, *P* = 0.002) (Fig 1). There were no statistical differences between groups for BCVA (F = 0.54, df = 2, *P* = 0.58), CMT (F = 315.4, df = 2, *P* = 0.85), ONLT (F = 1.7, df = 2, *P* = 0.181), or DDAF (F = 1.06, df = 2, *P* = 0.37). Regarding the patients who did not have any systemic findings, 7 of 10 were homozygous for p.(Met390Arg), 2 out of 10 were homozygous for p.(Arg160Gln), and 1 patient was heterozygous [(Met390Arg); p.(Asn524del)].

Patients who were homozygous for p.(Arg160Gln) had the earliest age of onset at the age of 7 and their first appointment by the age of 16. Heterozygous patients had a mean age of onset of 12 years and presented for the first appointment earlier than the other 2 groups, at a mean age of 15 years. In contrast, patients homozygous for p.(Met390Arg) showed the latest disease onset and baseline presentation with a mean age of 20.6 years and 32.1 years, respectively.

## Discussion

Herein, we present a retrospective observation cohort of patients with molecularly confirmed *BBS1* retinopathy. The clinical phenotype, imaging and electrophysiological features, and longitudinal evaluation are detailed; providing novel findings that improve genetic counseling, advice on prognosis, and have implications for clinical trial design and participant stratification. Seven *BBS1* variants were reported, and characteristic genotype–phenotype correlations were revealed.

Retinopathy has been reported as the most prevalent finding in patients with BBS across cohorts from different geographical areas, being present in up to 96% of the patients.^19^^,^^20^ Within our cohort, 20% of the patients had nonsyndromic retinopathy, which has been described in previous cohorts and case reports^21^^,^^22^ in ≥5 BBS-associated genes, including *BBS1.*

The most common systemic findings within our cohort were obesity and polydactyly, both present in 58.3% of the patients, followed by intellectual disability in 35.4% and renal abnormalities in 18.8% of the patients. Previous international cohorts^19^^,^^20^^,^^23^ have shown that the prevalence of polydactyly is usually higher in patients with BBS, ranging up to >90% in a recent report of *BBS1*-associated cases.^2^ Previous publications have shown that *BBS1* can be associated with an ocular and systemic phenotype that is less severe compared with other BBS genes.^24^ This could explain why, within our study, there is a low prevalence of systemic findings, with the exception of polydactyly and obesity.

In our cohort and a recent publication from Grudzinska et al, the age of onset for patients with *BBS1* was at the end of the second decade of life (18.1 and 19.4, respectively), whereas patients with disease associated with other BBS genes have been reported to have an age of onset that is usually within the first decade of life.^2^^,^^19^^,^^20^ The rate of loss of BCVA per year (0.05 LogMAR) was also lower compared with previous reports, where no distinction between genes was performed (0.09 LogMAR/year).^25^

The findings in this study describe a retinopathy, where the central retina is the earliest and most affected. On imaging, the first changes within the central macula on OCT imaging show the progression of central EZ loss in the subfoveal region, ranging from irregular EZ to loss of EZ and outer retina. Similarly, FAF findings showed an early hypoAF in the central macula and hypoAF changes in the nasal retina, which progress to a generalized retinal involvement with the appearance of hypoAF lacunae of atrophy in the central macula and pericentral retina.

This dataset characterizes the spectrum of electrophysiological retinal dysfunction and progression in *BBS1*-related retinal dystrophy. Retinal dysfunction ranges from mild to severe and is not predictable from *BBS1* genetic variant or age. Electroretinography is mostly in keeping with a rod–cone dystrophy (70%) or a similar degree of rod and cone dysfunction (22%). A cone–rod pattern of dysfunction, seen in 1 case monitored over 17 years, progressed to a rod–cone dystrophy, suggesting a greater rate of rod than cone dysfunction over time. Longitudinal ERG data spanning 4 to 19 years reveal a progressive decline in rod or rod and cono photoreceptor function in all cases. Macular dysfunction is usually but not always severe and not always predictable from the severity of full-field ERG abnormalities, highlighting the need for objective evaluation of both retinal and macular function.

Imaging and, especially, the electrophysiological findings during follow-up suggest that the retinal phenotype and the nature of retinal dysfunction in *BBS1* can depend on the stage of disease and the time since disease onset. Electrophysiological assessment provides a valuable insight into *BBS1*-related retinopathy, revealing a range of functional phenotypes with varying progression and macular involvement, not readily predictable from genotype or age alone, pertinent to future therapeutic intervention.

In previous reports, where p.(Arg160Gln) was described in a Turkish family and a White patient,^26^^,^^27^ it was referred to as a mild variant because they were nonsyndromic. However, in our study, patients who harbored a homozygous p.(Arg160Gln) variant, or 2 heterozygous variants, had an earlier age of onset and had their first appointment at a younger age compared with patients who carried a biallelic p.(Met390Arg) (20 years). *In silico* analysis suggested that p.(Arg160Gln) exerts a null effect through aberrant splicing, whereas p.(Met390Arg) is a missense variant, supporting a more severe molecular mechanism for p.(Arg160Gln). Therefore, although p.(Arg160Gln) has been described as mild in the context of BBS, in patients with biallelic *BBS1* retinopathy, it shows a more severe behavior (earlier onset and progression) compared with patients with p.(Met390Arg) (Fig 1).

It is well known that CMO is a common finding in patients with syndromic retinitis pigmentosa, such as Usher syndrome;^28^^,^^29^ interestingly, none of the patients developed CMO associated with their retinopathy through their follow-up, except for 3 patients who had intraretinal fluid due to CNV and retinal vein occlusion.

The p.(Met390Arg) missense represents the most common disease-causing variant in patients with *BBS1-*associated retinopathy, often in the homozygous state, and as shown in this study, these patients tend to have a broad window for treatment compared with patients affected with other BBS genes; therefore, they are good candidates for gene replacement therapy.

Knock-in mouse models (Bbs1^M390R/M390R^) have shown a severely disrupted morphology of photoreceptors involving the mislocation of rhodopsin in the ONL, and disorientation of outer segment membranous discs.^30^ Subretinal gene therapy with an adeno-associated virus vector (AAV5) in a BBS1 mouse model was found to rescue BBSome formation and rhodopsin localization. However, this study showed that overexpression of the BBS1 protein could be toxic, potentially due to an excess of 1 protein component disrupting normal BBSome assembly.^31^ Recently, Axovia Therapeutics developed an AAV9 vector expressing a codon-optimized human *BBS1* (hCOBBS1) designated AXV-101, which demonstrated therapeutic benefit in mouse models, with sustained ONLT and photoreceptor preservation at 6 months after subretinal injection. However, dosing must consider age-related and genotype-dependent differences in BBS1 expression, because wild-type animals may show artificial toxicity not relevant to patients.^32^ Axovia plan to undertake a first-in-human dose escalation phase I clinical trial for AXV-101 (ISRCTN96250868).

### Limitations

The retrospective nature of this study is associated with certain inherent limitations, such as variability in the available data, the lack of standardized protocols used for structural assessments, the lack of axial length measurements to correct transverse OCT and FAF measurements, and variable follow-up intervals. This could potentially influence our analysis because the progression could depend on the stage of the disease. Also, as our cohort were identified from retinal genetic clinics, there may be some degree of ascertainment bias, with all patients having retinopathy, and a possible overestimate of the overall proportion of patients with *BBS1*-associated disease, who have nonsyndromic retinopathy.

Future natural history studies could improve our predictions for patients with retinopathy associated with *BBS1* and create accurate outcomes for prospective clinical trials. The visual field data from our cohort was excluded from the analysis, which is known to be a useful functional assessment, because the information in the clinical records was minimal and from different platforms. Different genetic testing protocols were applied.

## Conclusions

This study examines the largest and longest follow-up longitudinal cohort of molecularly confirmed patients with *BBS1*-associated retinopathy. The ocular and systemic phenotype, detailed imaging, electrophysiological features, and disease progression are described, expanding the spectrum of functional and structural phenotypes related to *BBS1* variants.

Despite early involvement of the central retina, compared with other BBS genes, *BBS1* appears to have a relatively wide window for therapeutic intervention, especially in patients harboring the most common homozygous variant p.(Met390Arg). Also, given that many systemic features in most cases are noted since birth or early childhood, these patients can be diagnosed early. Prospective natural history studies are needed to further determine clinical outcomes.

## Supplementary Data

Figure S1

Supplemental Material Multimodal imaging

## References

[1] Dollfus H., Lilien M.R., Maffei P. (2024). Bardet-Biedl syndrome improved diagnosis criteria and management: Inter European Reference Networks consensus statement and recommendations. Eur J Hum Genet.

[2] Grudzinska Pechhacker M.K., Jacobson S.G., Drack A.V. (2021). Comparative natural history of visual function from patients with biallelic variants in BBS1 and BBS10. Invest Opthalmol Vis Sci.

[3] Moore S.J., Green J.S., Fan Y. (2005). Clinical and genetic epidemiology of Bardet-Biedl syndrome in Newfoundland: a 22-year prospective, population-based, cohort study. Am J Med Genet A.

[4] Esposito G., Testa F., Zacchia M. (2017). Genetic characterization of Italian patients with Bardet-Biedl syndrome and correlation to ocular, renal and audio-vestibular phenotype: identification of eleven novel pathogenic sequence variants. BMC Med Genet.

[5] Masek M., Etard C., Hofmann C. (2022). Loss of the Bardet-Biedl protein Bbs1 alters photoreceptor outer segment protein and lipid composition. Nat Commun.

[6] Lange C., Feltgen N., Junker B. (2009). Resolving the clinical acuity categories “hand motion” and “counting fingers” using the Freiburg Visual Acuity Test (FrACT). Graefes Archive Clin Exp Ophthalmol.

[7] Day A.C., Donachie P.H.J., Sparrow J.M., Johnston R.L. (2015). The Royal College of Ophthalmologists' National Ophthalmology Database study of cataract surgery: report 1, visual outcomes and complications. Eye.

[8] Georgiou M., Fujinami K., Vincent A. (2021). KCNV2-Associated retinopathy: detailed retinal phenotype and Structural Endpoints—KCNV2 Study Group report 2. Am J Ophthalmol.

[9] Sheck L.H.N., Esposti S.D., Mahroo O.A. (2021). Panel-based genetic testing for inherited retinal disease screening 176 genes. Mol Genet Genomic Med.

[10] Sergouniotis P.I., Chakarova C., Murphy C. (2014). Biallelic variants in TTLL5, encoding a tubulin glutamylase, cause retinal dystrophy. Am J Hum Genet.

[11] Carss K.J., Arno G., Erwood M. (2017). Comprehensive rare variant analysis via whole-genome sequencing to determine the molecular pathology of inherited retinal disease. The Am J Hum Genet.

[12] Hashem S.A., Georgiou M., Fujinami-Yokokawa Y. (2024). Genetics, clinical characteristics, and natural history of PDE6B-Associated retinal dystrophy. Am J Ophthalmol.

[13] Richards S., Aziz N., Bale S. (2015). Standards and guidelines for the interpretation of sequence variants: a joint consensus recommendation of the American College of Medical Genetics and Genomics and the Association for Molecular Pathology. Genet Med.

[14] Thompson D.A., Bach M., McAnany J.J. (2024). ISCEV standard for clinical pattern electroretinography (2024 update). Doc Ophthalmol.

[15] Robson A.G., Frishman L.J., Grigg J. (2022). ISCEV Standard for full-field clinical electroretinography (2022 update). Doc Ophthalmol.

[16] Robson A.G., Nilsson J., Li S. (2018). ISCEV guide to visual electrodiagnostic procedures. Doc Ophthalmol.

[17] (2000). Obesity: preventing and managing the global epidemic. Report of a WHO consultation. World Health Organ Tech Rep Ser.

[18] Scheidecker S., Hull S., Perdomo Y. (2015). Predominantly cone-system dysfunction as rare form of retinal degeneration in patients with molecularly confirmed Bardet-Biedl Syndrome. Am J Ophthalmol.

[19] Denniston A.K., Beales P.L., Tomlins P.J. (2014). Evaluation of visual function and NEEDS IN adult patients with BARDET–BIEDL syndrome. Retina.

[20] Beales P.L., Warner A.M., Hitman G.A. (1997). Bardet-Biedl syndrome: a molecular and phenotypic study of 18 families. J Med Genet.

[21] Weihbrecht K., Goar W.A., Pak T. (2017). Keeping an eye on Bardet-Biedl Syndrome: a comprehensive review of the role of Bardet-Biedl Syndrome genes in the eye. Med Res Arch.

[22] Fadaie Z., Whelan L., Dockery A. (2022). BBS1 branchpoint variant is associated with non-syndromic retinitis pigmentosa. J Med Genet.

[23] Nasser F., Kohl S., Kurtenbach A. (2022). Ophthalmic and genetic features of Bardet Biedl syndrome in a German cohort. Genes (Basel).

[24] Nasser F., Kohl S., Kurtenbach A. (2022). Ophthalmic and genetic features of bardet biedl syndrome in a German cohort. Genes (Basel).

[25] Fulton A.B. (1993). Natural course of visual functions in the Bardet-Biedl Syndrome. Arch Ophthalmol.

[26] Hichri H., Stoetzel C., Laurier V. (2005). Testing for triallelism: analysis of six BBS genes in a Bardet–Biedl syndrome family cohort. Eur J Hum Genet.

[27] Schmid F., Glaus E., Barthelmes D. (2011). U1 snRNA-mediated gene therapeutic correction of splice defects caused by an exceptionally mild BBS mutation. Hum Mutat.

[28] Romo-Aguas J.C., de Guimarães T.A.C., Kalitzeos A. (2025). Detailed Clinical, ophthalmic, and Genetic Characterization of MYO7A -Associated Usher Syndrome. Invest Ophthalmol Vis Sci Obesity: preventing and managing the global epidemic. Report of a WHO consultation. World Health Organ Tech Rep Ser.

[29] de Guimaraes T.A.C., Robson A.G., de Guimaraes I.M.C. (2024). CDH23 -Associated Usher Syndrome: clinical features, retinal imaging, and natural history. Invest Ophthalmol Vis Sci.

[30] Davis R.E., Swiderski R.E., Rahmouni K. (2007). A knockin mouse model of the Bardet–Biedl syndrome 1 M390R mutation has cilia defects, ventriculomegaly, retinopathy, and obesity. Proc Natl Acad Sci.

[31] Seo S., Mullins R.F., Dumitrescu A.V. (2013). Subretinal gene therapy of mice with Bardet-Biedl Syndrome type 1. Invest Opthalmology Vis Sci.

[32] Hernandez-Hernandez V., Elangkovan N., Freitas M. (2025). Expression levels of Bardet-Biedl Syndrome (BBS) genes change during retinal development showing an age depended dynamic regulation. Implications for determining gene therapies correct minimum effective doses (MEDs) and threshold safety levels for retinal gene therapies; the case of AXV-101 (BBS1). Invest Opthalmology Vis Sci.

