# Supplementary material for: Genetic and Phenotypic Characterization of a Large Cohort of Patients with BBS1-Retinopathy
Source: Ophthalmol Sci. 2026 Mar 19;6(5):101164. doi: 10.1016/j.xops.2026.101164 (PMC13098588; doi:10.1016/j.xops.2026.101164)
Supplement: Figure S1 [file mmc1.pdf]

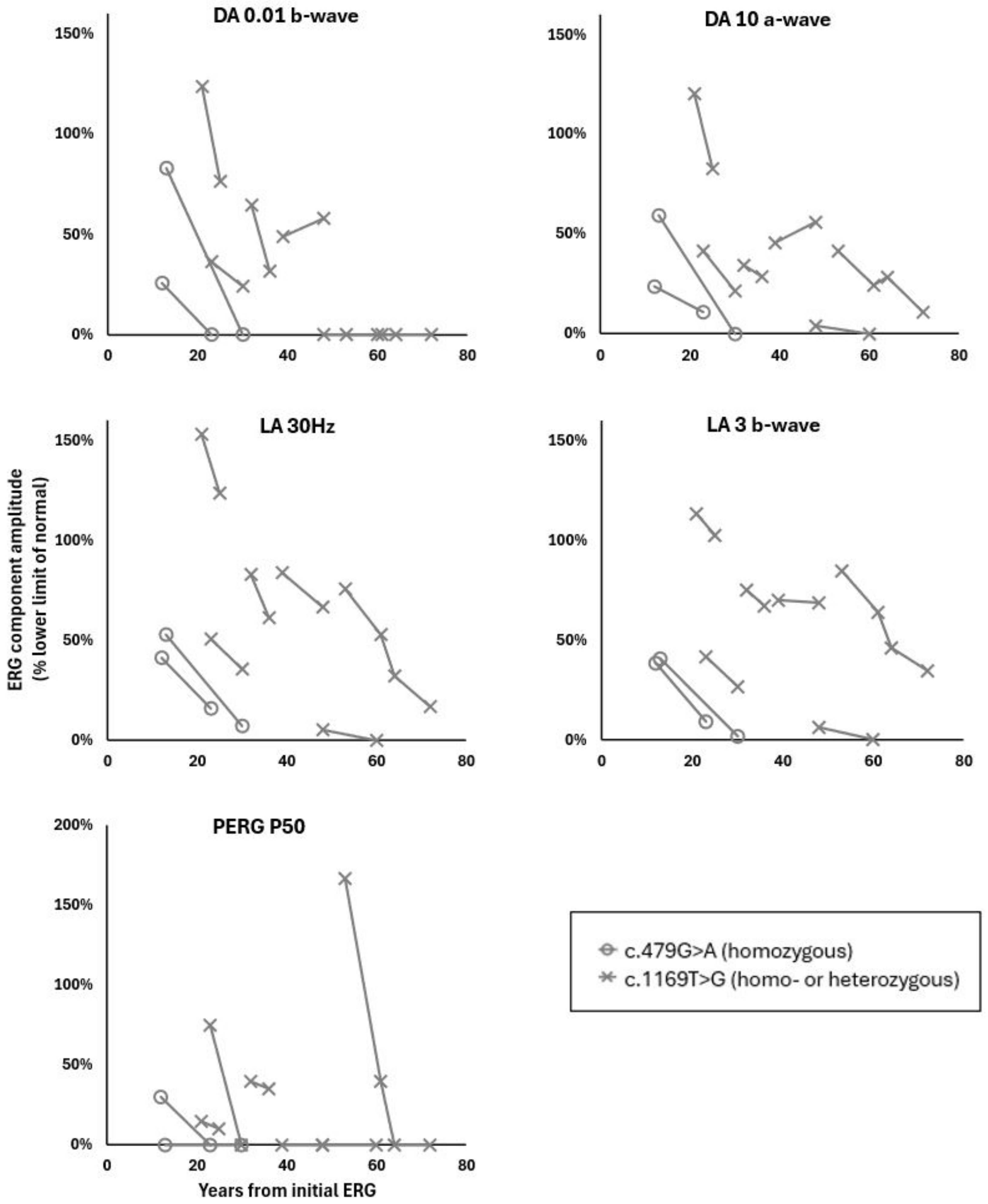

Supplemental Material Figure 1 ISCEV Standard ERG and PERG P50 amplitudes in 8 patients who underwent repeat testing. The ERG and PERG P50 component amplitudes are expressed as a percentage of the lower age-matched limit of normal, with each line showing the change over time for each patient. Symbols indicate BBS1 genetic variant.
