# Supplementary material for: Genetic and Phenotypic Characterization of a Large Cohort of Patients with BBS1-Retinopathy
Source: Ophthalmol Sci. 2026 Mar 19;6(5):101164. doi: 10.1016/j.xops.2026.101164 (PMC13098588; doi:10.1016/j.xops.2026.101164)
Supplement: Supplemental Material Multimodal imaging [file mmc2.pdf]

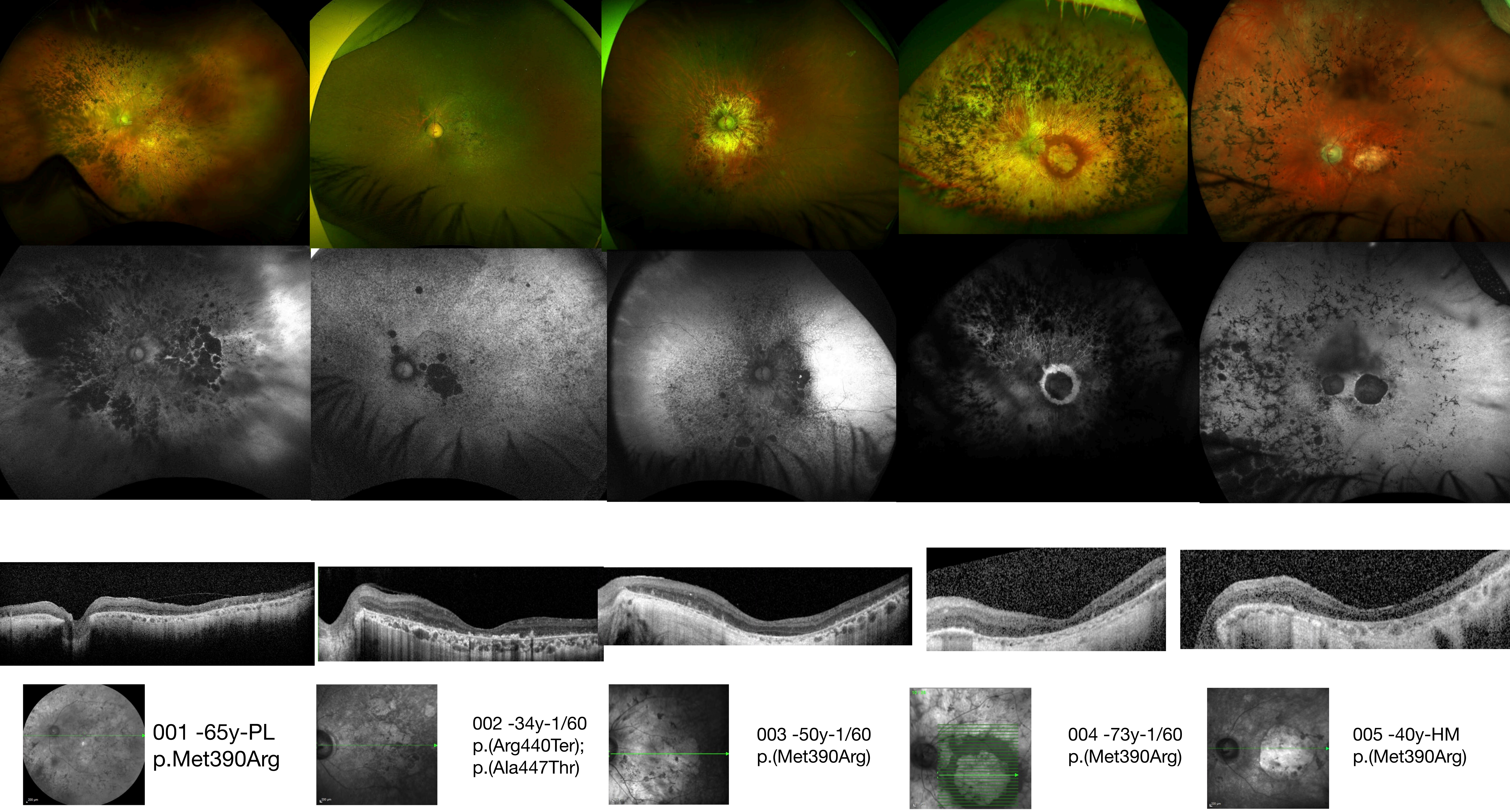
001 -65y-PL
p.Met390Arg
002 -34y-1/60
p.(Arg440Ter);
p.(Ala447Thr)
003 -50y-1/60
p.(Met390Arg)
004 -73y-1/60
p.(Met390Arg)
005 -40y-HM
p.(Met390Arg)

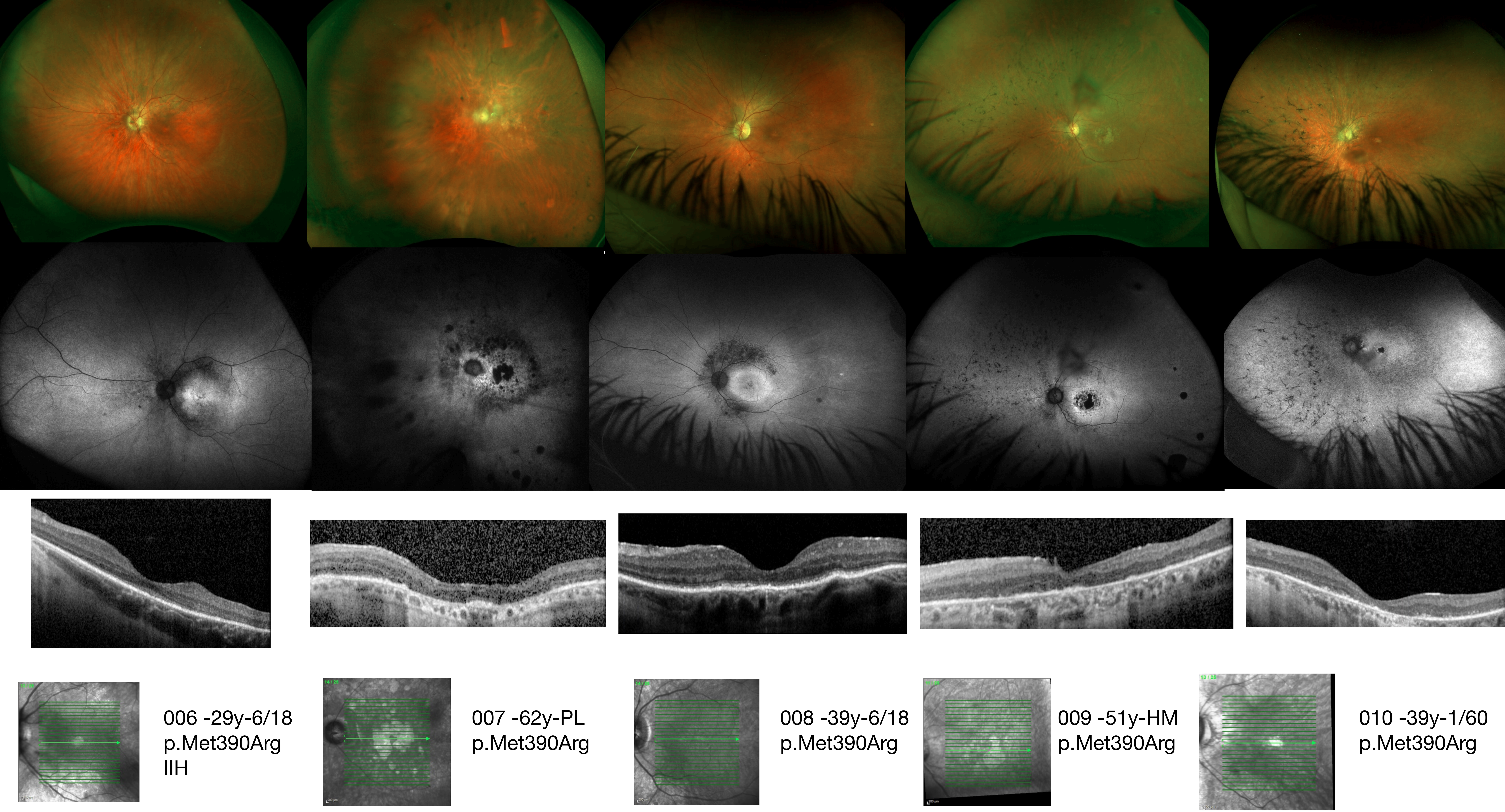
006 -29y-6/18
p.Met390Arg
IIH
007 -62y-PL
p.Met390Arg
008 -39y-6/18
p.Met390Arg
009 -51y-HM
p.Met390Arg
010 -39y-1/60
p.Met390Arg

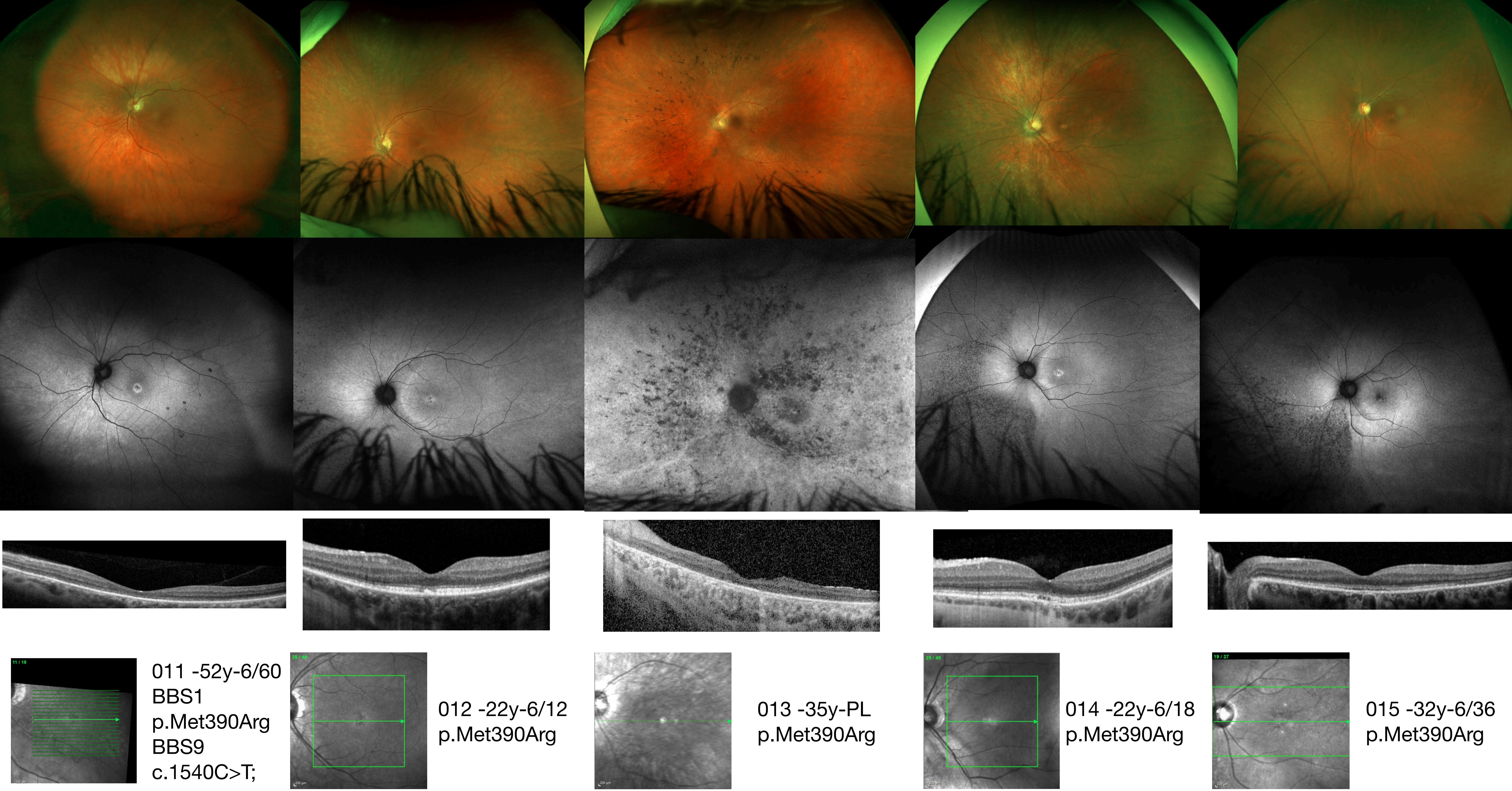
011 -52y-6/60
BBS1
p.Met390Arg
BBS9
c.1540C>T;
012 -22y-6/12
p.Met390Arg
013 -35y-PL
p.Met390Arg
014 -22y-6/18
p.Met390Arg
015 -32y-6/36
p.Met390Arg

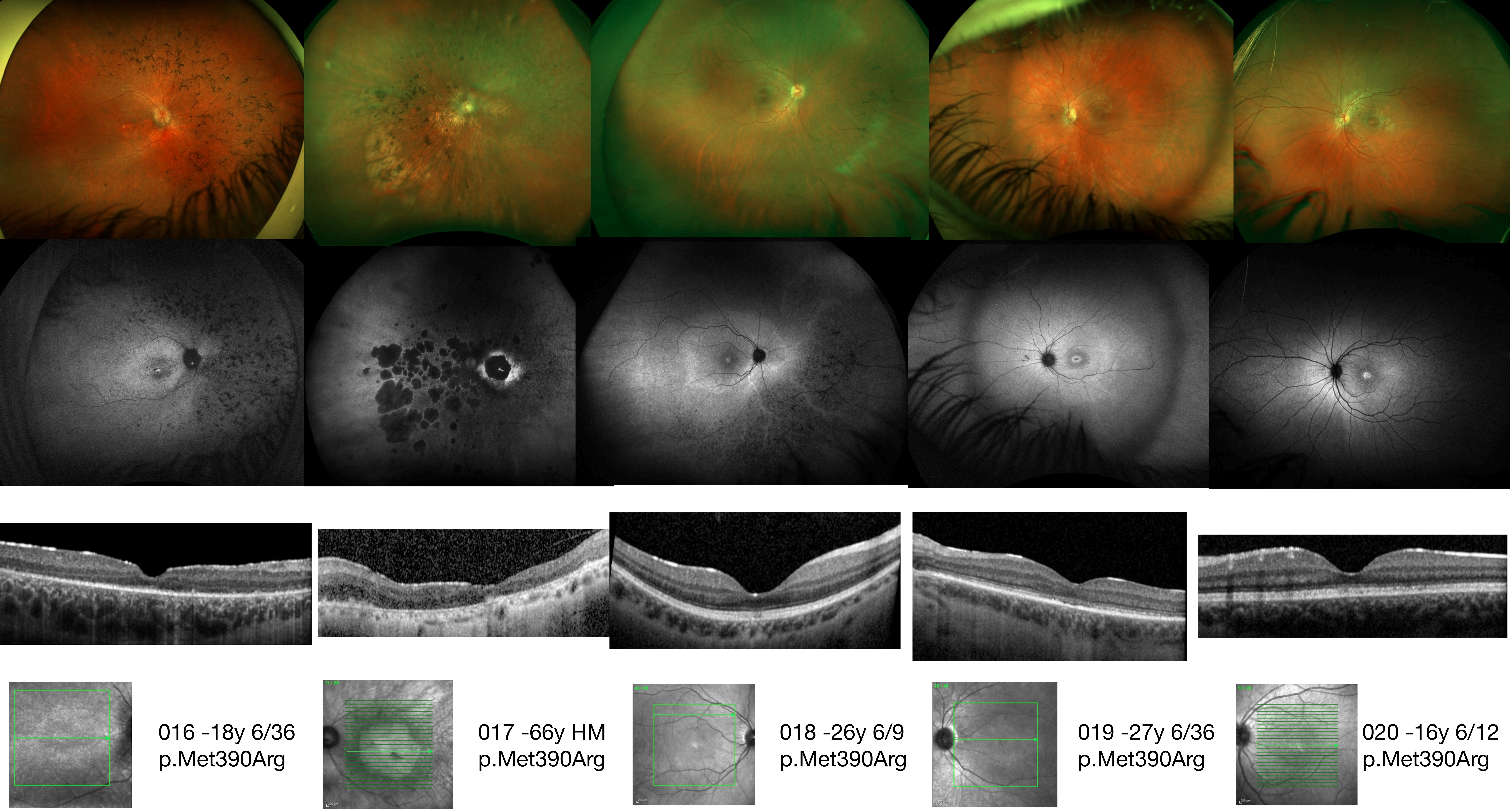
016 -18y 6/36
p.Met390Arg
017 -66y HM
p.Met390Arg
018 -26y 6/9
p.Met390Arg
019 -27y 6/36
p.Met390Arg
020 -16y 6/12
p.Met390Arg

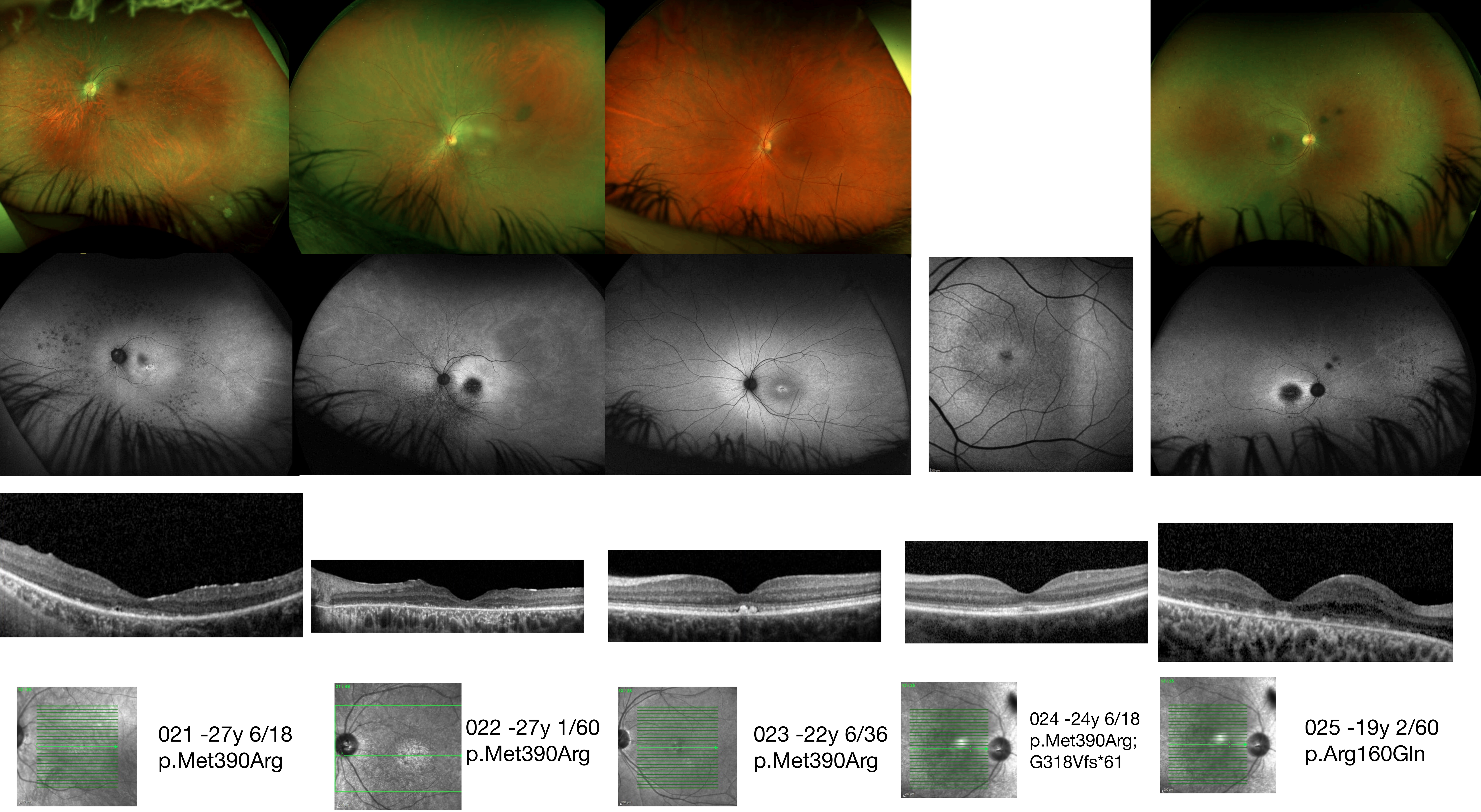

021 -27y 6/18
p.Met390Arg
022 -27y 1/60
p.Met390Arg
023 -22y 6/36
p.Met390Arg
024 -24y 6/18
p.Met390Arg;
G318Vfs*61
025 -19y 2/60
p.Arg160Gln

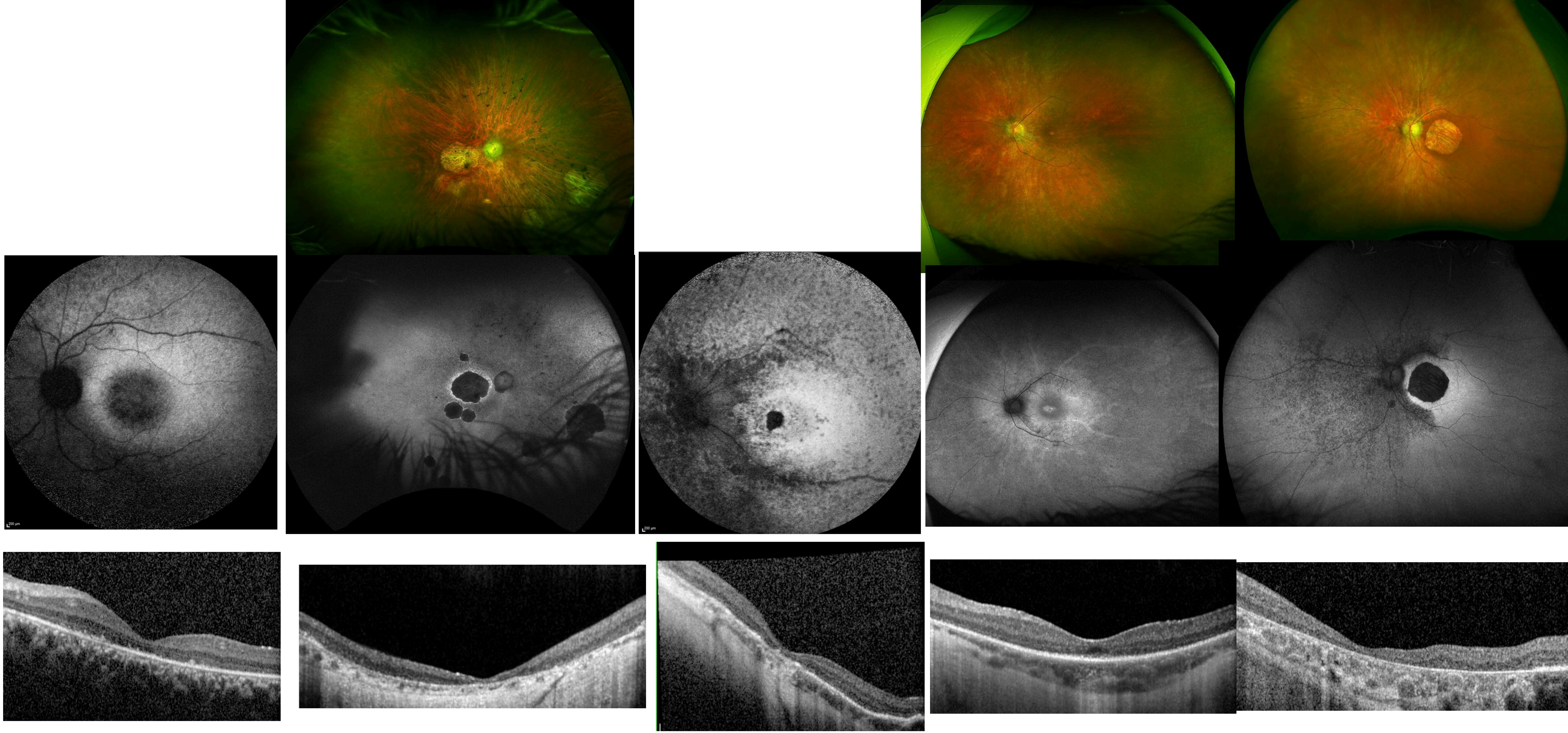

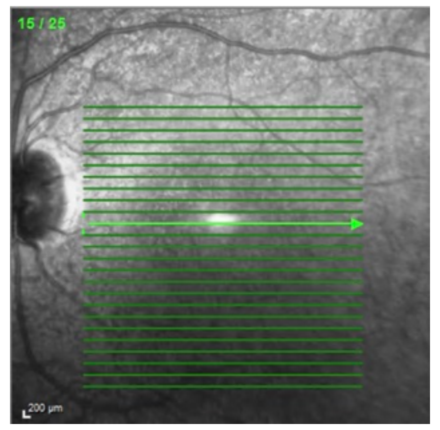

026 -18y 6/60
p.Arg160Gln

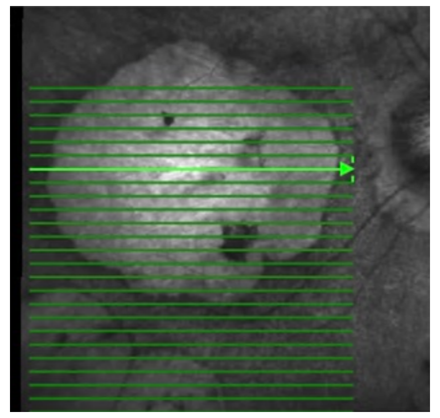

027 -53y HM
p.Met390Arg

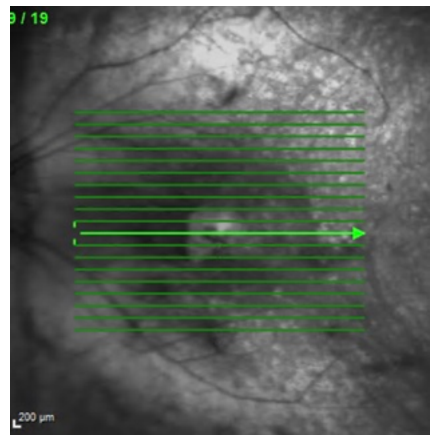

028 -52y 6/60
p.Met390Arg

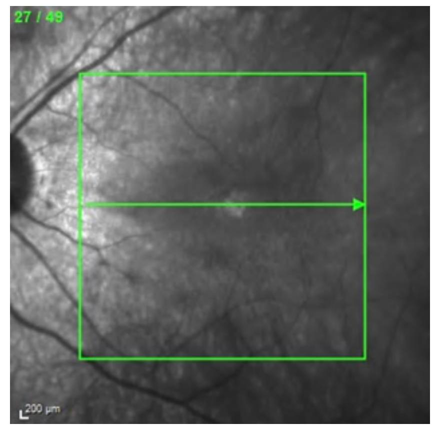

029 -20y 6/18
p.Met390Arg

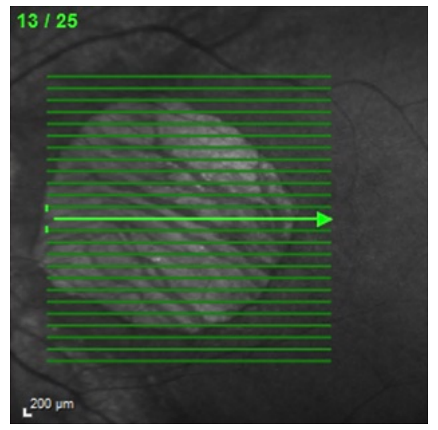

030 -54y 6/60
p.Met390Arg

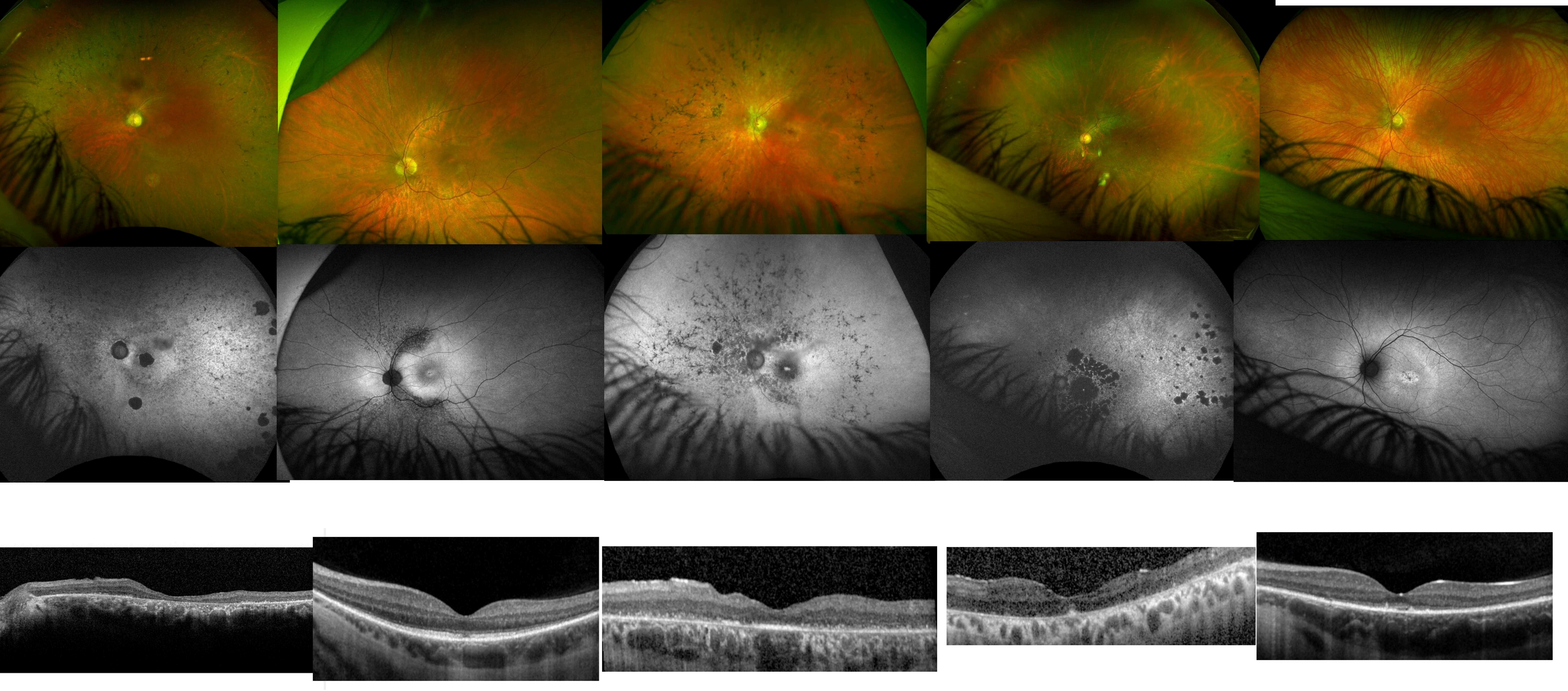

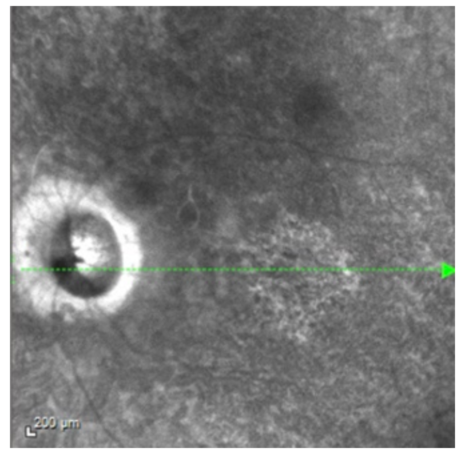

031 -31y 2/60
p.Met390Arg;
p.(Asn524del)

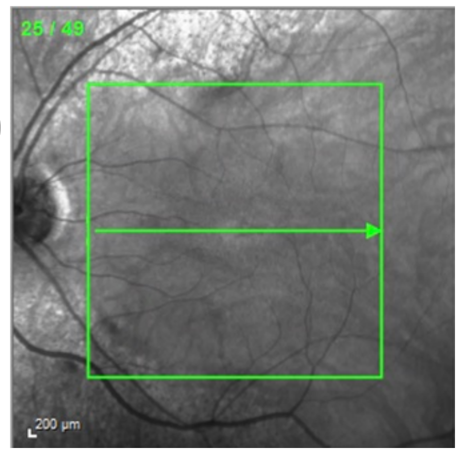

032 -29y 6/9
p.Met390Arg;

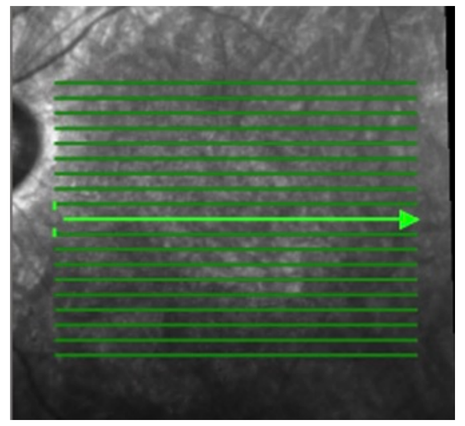

033 -31y 3/60
p.Met390Arg

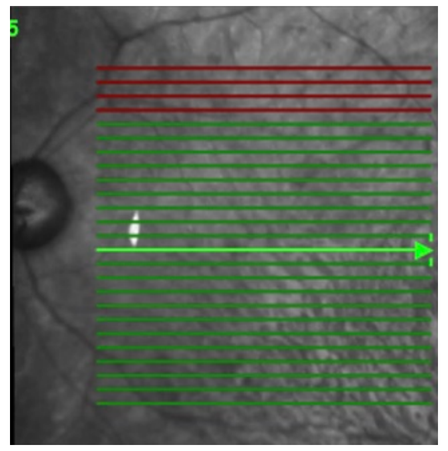

034 -33y HM
p.Arg160Gln

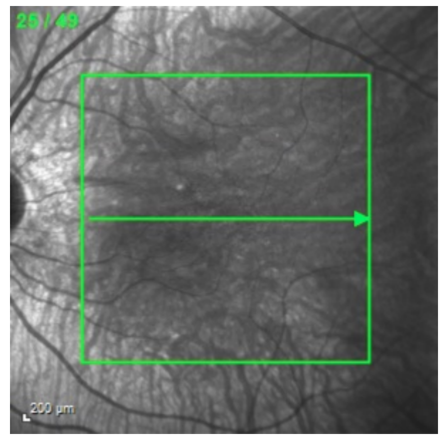

035 -22y 6/18
p.Met390Arg

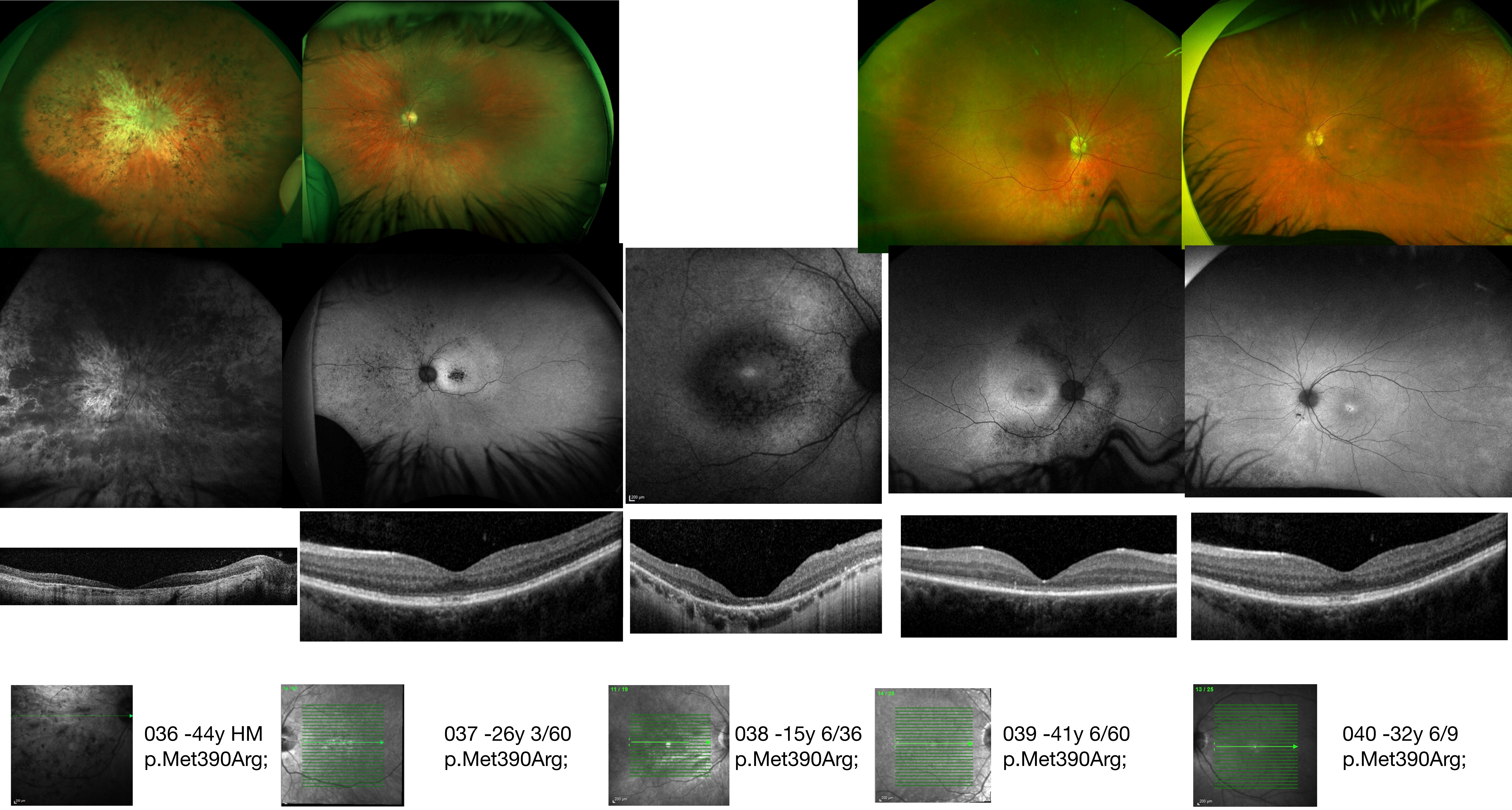
036 -44y HM
p.Met390Arg;
037 -26y 3/60
p.Met390Arg;
038 -15y 6/36
p.Met390Arg;
039 -41y 6/60
p.Met390Arg;
040 -32y 6/9
p.Met390Arg;

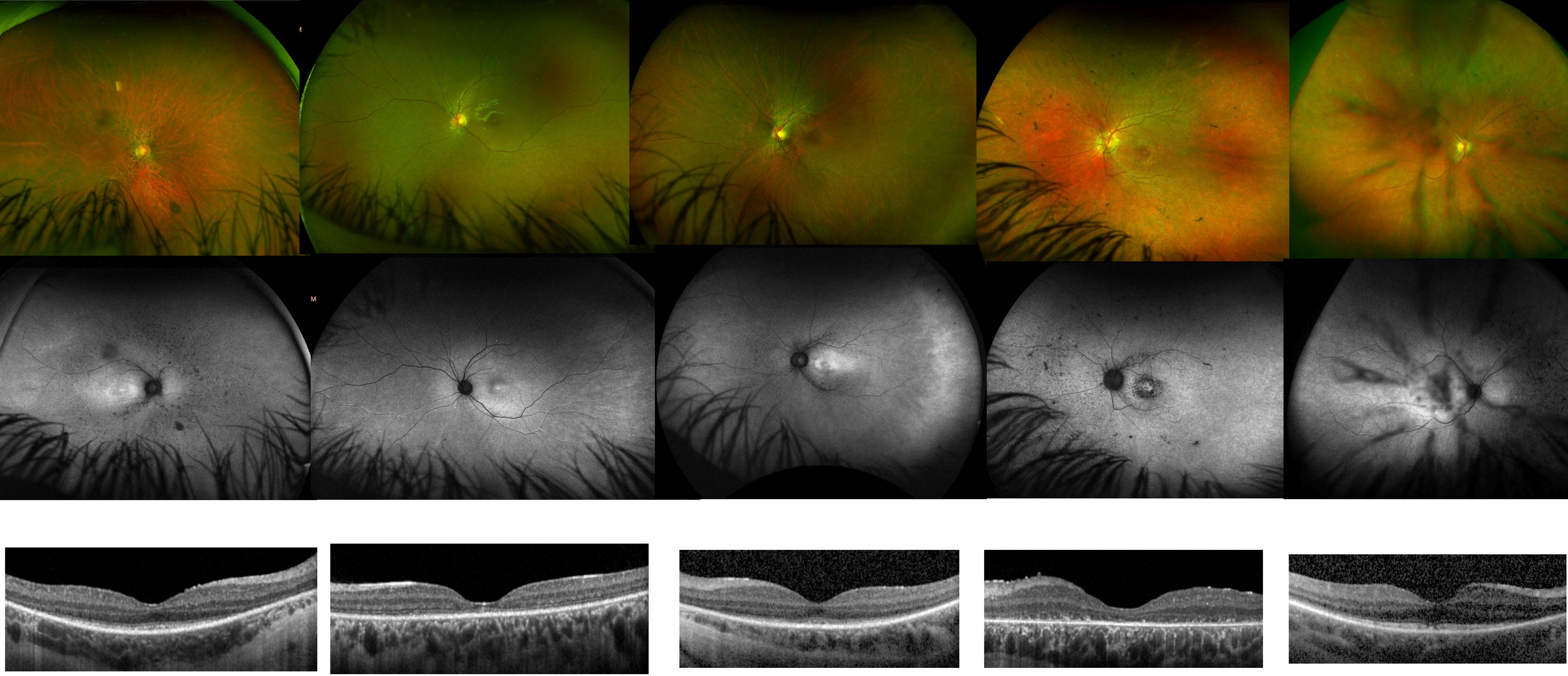

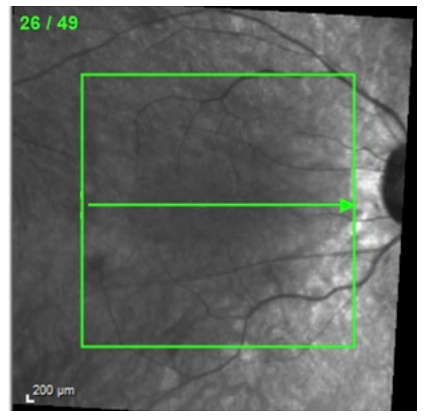

041 -23y 6/15
p.Met390Arg

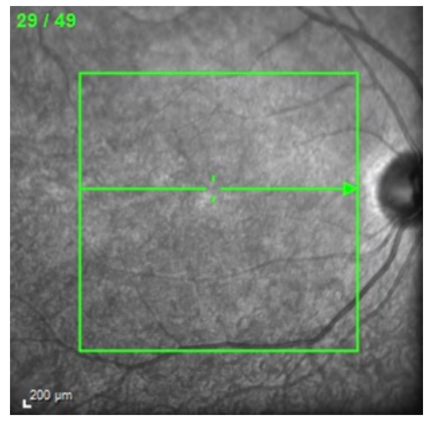

042 -13y- 6/18
p.Arg160Gln

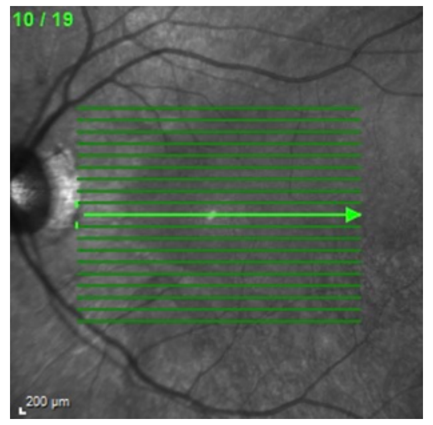

043 -23y 6/36
p.Arg160Gln

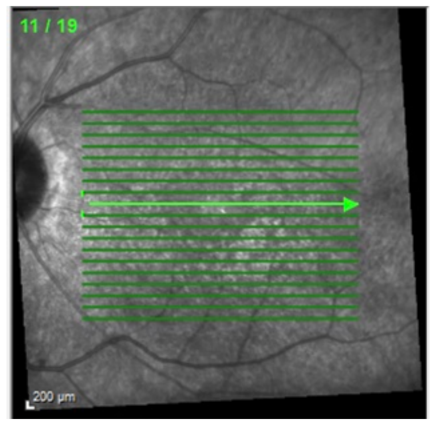

044 -20y 1/60
BBS1
p.Met390Arg;
p.Glu549Glyfs*9

BBS12
c.714dup
p.Arg239

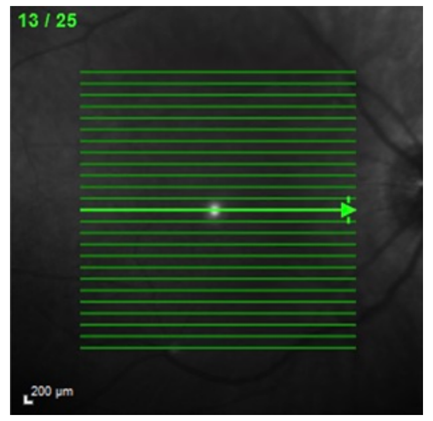

045 -23y 6/9
p.Met390Arg

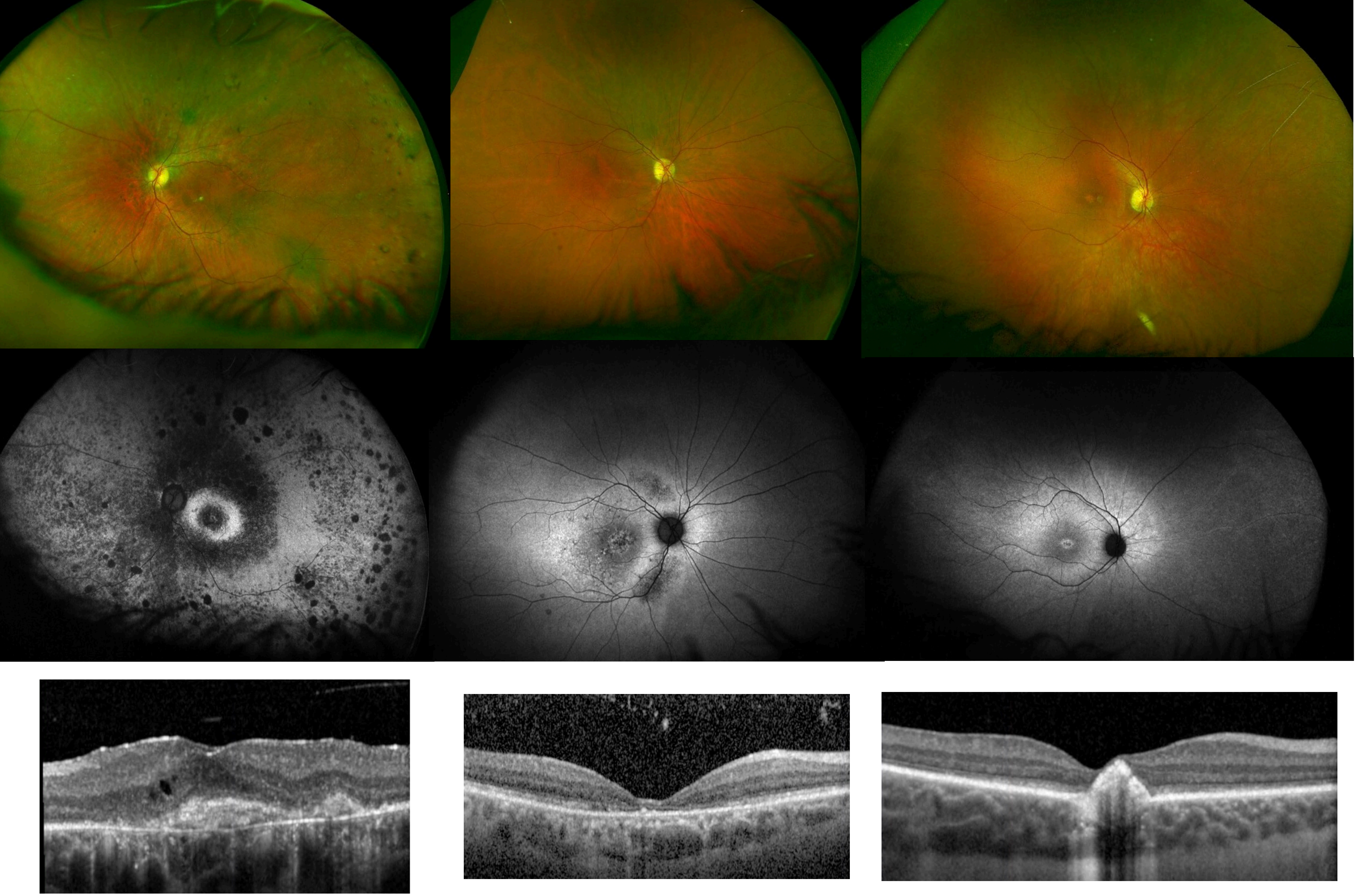

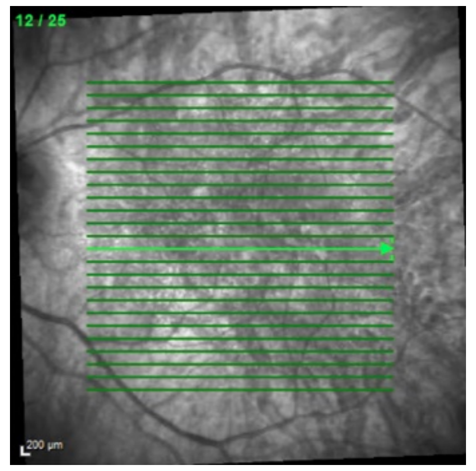

046 -67y 6/19
p.Met390Arg

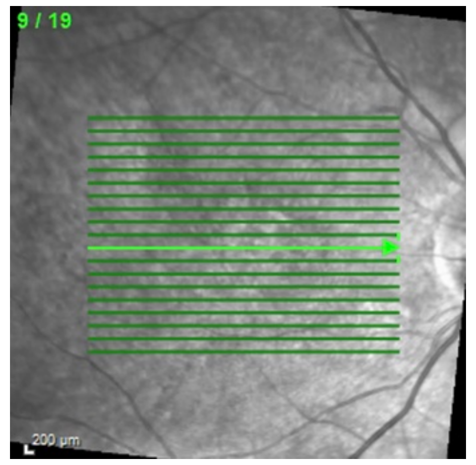

047 -52y-4/60
p.Met390Arg

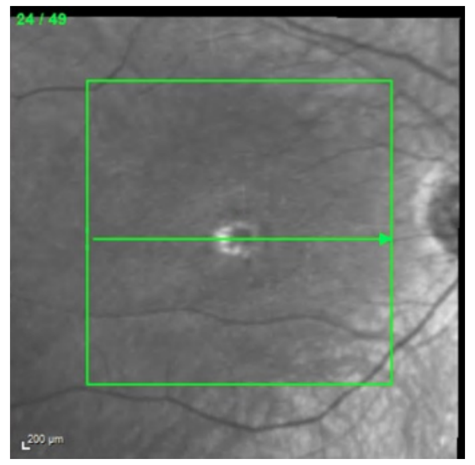

048 -13y - 6/24
p.Asn524del
p.Met390Arg
