# Supplementary material for: Genetic and Phenotypic Characterization of a Large Cohort of Patients with BBS1-Retinopathy
Source: Ophthalmol Sci. 2026 Mar 19;6(5):101164. doi: 10.1016/j.xops.2026.101164 (PMC13098588; doi:10.1016/j.xops.2026.101164)
Supplement: Table S1 [file mmc3.pdf]

**Supplemental Table 1. Detailed results of in silico molecular genetic assessment of 7 detected *BBS1* variants.**

| Variant No | Patient/family number | Nucleotide change (NM_024649.5) | Amino acid change (NP_078925.3) | Position (hg38) | Coding impact | Location | dbSNP ID | ClinVar ID | Segregation | ACMG Classification | | | | | GnomAD allele frequency | |
|---|---|---|---|---|---|---|---|---|---|---|---|---|---|---|---|---|
| | | | | | | | | | | Verdict | Idntified classification rules | | | | Allele frequency (exome) | |
| | | | | | | | | | | | Factor 1 | Factor 2 | Factor 3 | Factor 4 | East Asian | South Asian |
| 1 | 6 patients | c.479G>A | p.Arg160Gln | 66515586 | splice_site_alteration/missense_variant | exon 5 of 17 position 47 of 47 (splicing-ACMG, splicing, coding) | rs376894444 | 370228 | Homo | Pathogenic | PVS1 | PM2 | PM3 | PP5 | 0 | 0 |
| 2 | 1 patient | c.951+58C>T | p.Gly318ValfsTer61 | 66523634 | splice_site_alteration/intron_variant | intron 10 of 16 position 58 of 147 (intronic) | rs1856346961 | 917920 | Het, may be no | Likely pathogenic | PVS1_moderate | PM2 | PM3 | PP5 | 0 | 0 |
| 3 | 41 patients | c.1169T>G | p.Met390Arg | 66526181 | missense_variant | exon 12 of 17 position 59 of 70 (coding) | rs113624356 | 12143 | Homo | Pathogenic | PS1 | PS3 | PM3 | PP5 | 0 | 0 |
| 4 | 1 patient | c.1318C>T | p.Arg440Ter | 66526786 | stop_gained | exon 13 of 17 position 138 of 159 (coding, NMD) | rs1014835928 | 551887 | Het, may be no | Pathogenic | PVS1 | PM2 | PM3 | PP5 | 0 | 0 |
| 5 | 1 patient | c.1339G>A | p.Ala447Thr | 66526807 | splice_site_alteration/missense_variant | exon 13 of 17 position 159 of 159 (splicing-ACMG, splicing, coding) | rs200116631 | 531824 | Het, may be no | Likely pathogenic | PVS1_moderate | PM2 | PM3 | PP5 | 0.000151134 | 0.000173897 |
| 6 | 2 patients | c.1570_1572del | p.Asn524del | 66530988 | inframe_deletion | exon 15 of 17 position 97-99 of 135 (coding) | rs863224782 | 216741 | Het, may be no | Likely pathogenic | PM2 | PM3 | PM4 | PP5 | 0 | 0 |
| 7 | 1 patient | c.1643dup | p.Glu549GlyfsTer9 | 66531690 | frameshift_variant | exon 16 of 17 before position 36 of 87 (coding) | rs773632109 | 371262 | Het, may be no | Likely pathogenic | PVS1 | PM2 | PP5 | | 0 | 0 |

Supplemental Material Table 1. Variant analysis

A combination of Sanger direct sequencing and next-generation sequencing, including a panel of retinal dystrophy genes, whole exome sequencing, and whole genome sequencing, was used to identify variants in BBS1. All recruited patients were reassessed for their detected BBS1 variants (BBS1: Refseq Reference: NM_024649.5; NP_078925.3; Emsembl transcript ID: ENST00000318312.12; UniProtKB: Q8NFJ9).Sequence variant nomenclature was obtained according to the guidelines of the Human Genome Variation Society (HGVS) by using Mutalyzer 2.0. Classification of all detected variants was also performed based on the guidelines of the American College of Medical Genetics and Genomics (ACMG).

| | | | | | | | | | | | | | | | | General prediscion | | | | |
|---|---|---|---|---|---|---|---|---|---|---|---|---|---|---|---|---|---|---|---|---|
| | | | | | | Allele frequency (genome) | | | | | | | Coverage in gnomAD Exomes samples | | | MutationTaster | | | FATHMM | |
| African | European (Non-Finnish) | Admixed American | Total | POPMAX Alt Allele Freq (AF) | POPMAX | East Asian | Admixed American | African | European (Non-Finnish) | Total | POPMAX Alt Allele Freq (AF) | POPMAX | Mean coverage | Median coverage | % of samples over 20x coverage | Prediction | Accuruacy | Converted rankscore | Prediction | Score |
| 5.97372e-05 | 3.59715e-06 | 0 | 7.5246e-06 | 0.00017337 | mid | 0 | 0 | 9.6609e-05 | 0 | 2.62964e-05 | afr | afr | 46.4 | 33 | 99.94% | Disease Causing | 0.999999 | 0.81001 | Damaging | -2.79 |
| 0 | 4.49658e-06 | 0 | 4.1088e-06 | 4.49658e-06 | nfe | NA | NA | NA | NA | NA | NA | NA | 31.1 | 31 | 99.29% | NA | NA | NA | NA | NA |
| 0.000477926 | 0.00337775 | 0.00100617 | 0.0027431 | 0.00337775 | nfe | 0 | 0.0026154 | 0.000889851 | 0.00289544 | 0.00204821 | nfe | nfe | 32 | 31 | 99.78% | Disease Causing Automatic | 0.944844 | 0.81001 | Damaging | 0.45 |
| 0 | 2.69781e-06 | 2.23594e-05 | 2.73618e-06 | 2.23594e-05 | amr | 0 | 0 | 0 | 1.46972e-05 | 6.5703e-06 | NA | nfe | 32.7 | 32 | 99.98% | Disease Causing Automatic | 1 | 0.81001 | NA | NA |
| 0 | 8.99271e-06 | 0 | 2.18894e-05 | 0.000173897 | sas | 0.000192827 | 0 | 4.81e-05 | 4.41073e-05 | 4.59547e-05 | nfe | sas | 32.6 | 32 | 99.98% | Disease Causing | 1 | 0.81001 | Damaging | -4.09 |
| 0 | 9.89198e-06 | 0 | 7.52449e-06 | 9.89198e-06 | nfe | 0 | 6.54108e-05 | 0 | 1.46985e-05 | 1.31401e-05 | NA | amr | 57.7 | 32 | 99.99% | NA | NA | NA | NA | NA |
| 0 | 1.07913e-05 | 0 | 8.20861e-06 | 1.07913e-05 | nfe | NA | NA | NA | NA | NA | NA | NA | 51.1 | 32 | 99.98% | NA | NA | NA | NA | NA |

| | | | | | | | | | | | Functional prediction | | | | | | | |
|---|---|---|---|---|---|---|---|---|---|---|---|---|---|---|---|---|---|---|
| | FATHMM MKL | | | FATHMM XF | | | CADD | REVEL | | | SIFT | | | PROVEAN | | | Polyphen2 | |
| Converted rankscore | Prediction | Score | Converted rankscore | Prediction | Score | Converted rankscore | Score | Prediction Cut off = 0.5 | Score | Rankscore | Prediction | Score | Converted rankscore | Prediction | Score | Converted rankscore | Prediction | Score |
| 0.91019 | Damaging | 0.99407 | 0.95634 | Damaging | 0.924163 | 0.90177 | 6.99682 | Likely disease causing | 0.599 | 0.84128 | Damaging | 0.134 | 0.56456 | Neutral | -2.23 | 0.51811 | Probably damaging | 0.947 |
| NA | NA | NA | NA | NA | NA | NA | 1.20156 | NA | NA | NA | NA | NA | NA | NA | NA | NA | NA | NA |
| 0.89953 | Damaging | 0.98651 | 0.85151 | Damaging | 0.752736 | 0.69314 | 3.87497 | Likely disease causing | 0.662 | 0.87381 | Damaging | 0.012 | 0.54683 | Damaging | -4.05 | 0.75537 | Possibly damaging | 0.347 |
| NA | Damaging | 0.86805 | 0.46167 | Neutral | 0.228155 | 0.35186 | 7.06641 | NA | NA | NA | NA | NA | NA | ? | NA | NA | ? | NA |
| 0.96826 | Damaging | 0.94494 | 0.61346 | Damaging | 0.509783 | 0.5391 | 4.36929 | Likely benign | 0.347 | 0.66863 | Tolerated | 0.556 | 0.20683 | Neutral | -0.22 | 0.10656 | Benign | 0.02 |
| NA | NA | NA | NA | NA | NA | NA | 1.73234 | NA | NA | NA | NA | NA | NA | NA | NA | NA | NA | NA |
| NA | NA | NA | NA | NA | NA | NA | 4.01192 | NA | NA | NA | NA | NA | NA | NA | NA | NA | NA | NA |

| Human Splice Finder 3.0 | Conservation | | | | Conservation | | | | Report name | Year | Report name | Year |
|---|---|---|---|---|---|---|---|---|---|---|---|---|
| | PhyloP30way | | PhastCons30way | | PhyloP100way | | PhastCons100way | | | | | |
| | Mammalian | Mammalian rankscore | Mammalian | Mammalian rankscore | vertebrate | vertebrate rankscore | vertebrate | vertebrate rankscore | | | | |
| Broken WT Donor Site : Alteration of the WT Donor site, most probably affecting splicing | 1.175 | 0.73717 | 0.996 | 0.59523 | 8.027 | 0.89153 | 1 | 0.71638 | Combining Engineered U1 snRNA and Antisense Oligonucleotides to Improve the Treatment of a BBS1 Splice Site Mutation | 2019 | Testing for triallelism: analysis of six BBS genes in a Bardet–Biedl syndrome family cohort | 2005 |
| New Donor splice site : Activation of a cryptic Donor site. Potential alteration of splicing | NA | NA | NA | NA | NA | NA | NA | NA | The Clinical and Mutational Spectrum of Bardet–Biedl Syndrome in Saudi Arabia | 2024 | Predominantly Cone-System Dysfunction as Rare Form of Retinal Degeneration in Patients With Molecularly Confirmed Bardet-Biedl Syndrome | 2015 |
| New Acceptor splice site : Activation of a cryptic Acceptor site. Potential alteration of splicing | 1.138 | 0.64695 | 1 | 0.86279 | 4.696 | 0.6146 | 1 | 0.71638 | Bardet-Biedl syndrome improved diagnosis criteria and management: Inter European Reference Networks consensus statement and recommendations | 2024 | Spectrum of Genetic Variants in the Most Common Genes Causing Inherited Retinal Disease in a Large Molecularly Characterized United Kingdom Cohort | 2024 |
| New Donor splice site : Activation of a cryptic Donor site. Potential alteration of splicing | 1.022 | 0.39869 | 1 | 0.86279 | 0.869 | 0.27648 | 1 | 0.71638 | Spectrum of pathogenic variants and high prevalence of pathogenic BBS7 variants in Russian patients with Bardet–Biedl syndrome | 2024 | | |
| Broken WT Donor Site : Alteration of the WT Donor site, most probably affecting splicing | 1.172 | 0.72986 | 0.66 | 0.30079 | 5.18 | 0.64886 | 1 | 0.71638 | Whole exome sequencing uncovered highly penetrant recessive mutations for a spectrum of rare genetic pediatric diseases in Bangladesh | 2021 | Case Report: Identification Pathogenic Abnormal Splicing of BBS1 Causing Bardet–Biedl Syndrome Type I (BBS1) due to Missense Mutation | 2022 |
| New Acceptor splice site : Activation of a cryptic Acceptor site. Potential alteration of splicing (HSF)<br>New Donor splice site : Activation of a cryptic Donor site. Potential alteration of splicing (HSF) | NA | NA | NA | NA | NA | NA | NA | NA | Extensive Phenotyping for Potential Weight-Inducing Factors in an Outpatient Population with Obesity | 2019 | | |
| New Acceptor splice site : Activation of a cryptic Acceptor site. Potential alteration of splicing (cryptic exon activation) | NA | NA | NA | NA | NA | NA | NA | NA | Spectrum of Genetic Variants in the Most Common Genes Causing Inherited Retinal Disease in a Large Molecularly Characterized United Kingdom Cohor | 2024 | | |
