# Supplementary material for: Genetic and Phenotypic Characterization of a Large Cohort of Patients with BBS1-Retinopathy
Source: Ophthalmol Sci. 2026 Mar 19;6(5):101164. doi: 10.1016/j.xops.2026.101164 (PMC13098588; doi:10.1016/j.xops.2026.101164)
Supplement: Table S2 [file mmc4.pdf]

				Variant1		Variant 2		
Patient ID	Family number		Homozygous (Y/N)	c.DNA	Protein	c.DNA	Protein	Other variants
MEH 001	16383		Yes	c.1169T>G	p.(Met390Arg)			
MEH 002	26882		No	c.1339G>A	p.(Ala447Thr)	c.1318C>T	p.(Arg440Ter)	
MEH 003	18815		Yes	c.1169T>G	p.(Met390Arg)			
MEH 004	16086		Yes	c.1169T>G	p.(Met390Arg)			
MEH 005	21219		Yes	c.1169T>G	p.(Met390Arg)			
MEH 006	26416		Yes	c.1169T>G	p.(Met390Arg)			
MEH 007	26359		Yes	c.1169T>G	p.(Met390Arg)			
MEH 008	26719		Yes	c.1169T>G	p.(Met390Arg)			
MEH 009	5408		Yes	c.1169T>G	p.(Met390Arg)			
MEH 010	20381		Yes	c.1169T>G	p.(Met390Arg)			
MEH 011	19682		Yes	c.1169T>G	p.(Met390Arg)			BBS9 - c.1540C>T, p.(Arg514*)
MEH 012	29929		Yes	c.1169T>G	p.(Met390Arg)			
MEH 013	4965		Yes	c.1169T>G	p.(Met390Arg)			
MEH 014	29935		Yes	c.1169T>G	p.(Met390Arg)			
MEH 015	21517		Yes	c.1169T>G	p.(Met390Arg)			
MEH 016	29744		Yes	c.1169T>G	p.(Met390Arg)			
MEH 017	20412		Yes	c.1169T>G	p.(Met390Arg)			
MEH 018	26193		Yes	c.1169T>G	p.(Met390Arg)			
MEH 019	28874		Yes	c.1169T>G	p.(Met390Arg)			
MEH 020	26529		Yes	c.1169T>G	p.(Met390Arg)			
MEH 021	25807		Yes	c.1169T>G	p.(Met390Arg)			
MEH 022	16999		Yes	c.479G>A	p.(Arg160Gln)			
MEH 023	24786		Yes	c.1169T>G	p.(Met390Arg)			
MEH 024	17679		No	c.1169T>G	p.(Met390Arg)	c.953+58C>T	p.(G318Vfs*61)	
MEH 025	22087		Yes	c.479G>A	p.(Arg160Gln)			
MEH 026	22087		Yes	c.479G>A	p.(Arg160Gln)			
MEH 027	3341		Yes	c.1169T>G	p.(Met390Arg)			
MEH 028	19643		Yes	c.1169T>G	p.(Met390Arg)			
MEH 029	31241		Yes	c.1169T>G	p.(Met390Arg)			
MEH 030	19521		Yes	c.1169T>G	p.(Met390Arg)			
MEH 031	3824		No	c.1169T>G	p.(Met390Arg)	c.1570_1572del	p.(Asn524del)	
MEH 032	29651		Yes	c.1169T>G	p.(Met390Arg)			
MEH 033	21155		Yes	c.1169T>G	p.(Met390Arg)			
MEH 034	15996		Yes	c.479G>A	p.(Arg160Gln)			
MEH 035	28008		Yes	c.1169T>G	p.(Met390Arg)			
MEH 036	19422		Yes	c.1169T>G	p.(Met390Arg)			
MEH 037	26089		Yes	c.1169T>G	p.Met390Arg			
MEH 038	20977		Yes	c.1169T>G	p.(Met390Arg)			
MEH 039	23250		Yes	c.1169T>G	p.(Met390Arg)			
MEH 040	25279		Yes	c.1169T>G	p.(Met390Arg)			
MEH 041	30516		Yes	c.1169T>G	p.(Met390Arg)			
MEH 042	28815		Yes	c.479G>A	p.(Arg160Gln)			
MEH 043	17339		No	c.479G>A	p.(Arg160Gln)			
MEH 044	24255		No	c.1169T>G	p.(Met390Arg)	c.1643dup	p.Glu549Glyfs*9	BBS12 - c.714dup, p.(Arg239*)
MEH 045	26471		Yes	c.1169T>G	p.(Met390Arg)			
MEH 046	19214		Yes	c.1169T>G	p.(Met390Arg)			
MEH 047	18182		Yes	c.1169T>C	p.(Met390Arg)			
MEH 048	28228		No	c.1169T>G	p.(Met390Arg)	c.1570_1572delAAC	p.(Asn524del)	

Supplemental Material Table 2 - Subjects genotype
