# Supplementary material for: Genetic and Phenotypic Characterization of a Large Cohort of Patients with BBS1-Retinopathy
Source: Ophthalmol Sci. 2026 Mar 19;6(5):101164. doi: 10.1016/j.xops.2026.101164 (PMC13098588; doi:10.1016/j.xops.2026.101164)
Supplement: Supplemental material methods [file mmc5.pdf]

Supplemental material Methods

In silico molecular genetic analysis was conducted. Minor allele frequency for the identified variants in the general population was assessed in the Genome Aggregation Database (gnomAD, version 4.0) datasets. The population data and general coverage by whole exome sequence were also provided with the gnomAD database. General prediction scores were further calculated using MutationTaster, FATHMM, CADD, and REVEL. Functional prediction was performed employing SIFT, PROVEAN, and Polyphen 2. Human splicing finder 3.0 was applied for splicing defects prediction. Mammalian (PhyloP30way and PhastCons30way) and vertebrate (PhyloP100way and PhastCons100way) conservation were also investigated. The previously reported variants were surveyed with the HGMD database and ClinVar database (accessed on August 2025).
